# Evaluation of Angiogenesis-Related Genes as Prognostic Biomarkers of Bevacizumab Treated Ovarian Cancer Patients: Results from the Phase IV MITO16A/ManGO OV-2 Translational Study

**DOI:** 10.3390/cancers13205152

**Published:** 2021-10-14

**Authors:** Daniela Califano, Daniela Gallo, Gian Luca Rampioni Vinciguerra, Rossella De Cecio, Laura Arenare, Simona Signoriello, Daniela Russo, Gabriella Ferrandina, Francesca Citron, Nunzia Simona Losito, Piera Gargiulo, Vittorio Simeon, Giovanni Scambia, Sabrina Chiara Cecere, Marco Montella, Nicoletta Colombo, Germana Tognon, Eliana Bignotti, Gian Franco Zannoni, Vincenzo Canzonieri, Alessandra Ciucci, Anna Spina, Giosuè Scognamiglio, Michele Del Sesto, Clorinda Schettino, Maria Carmela Piccirillo, Francesco Perrone, Paolo Chiodini, Sandro Pignata, Gustavo Baldassarre

**Affiliations:** 1 Microenvironment Molecular Targets Unit, Istituto Nazionale Tumori IRCCS, Fondazione G. Pascale, 80131 Napoli, Italy; dcalifano@yahoo.com (D.C.); d.russo@istitutotumori.na.it (D.R.); a.spina@istitutotumori.na.it (A.S.); 2 Department of Woman and Child Health, Fondazione Policlinico Universitario A. Gemelli IRCCS, 00168 Roma, Italy; daniela.gallo@unicatt.it (D.G.); gabriella.ferrandina@gmail.com (G.F.); giovanni.scambia@policlinicogemelli.it (G.S.); gianfranco.zannoni@unicatt.it (G.F.Z.); Alessandra.ciucci@unicatt.it (A.C.); 3 Molecular Oncology Unit, Centro di Riferimento Oncologico di Aviano (CRO), IRCCS, National Cancer Institute, 33081 Aviano, Italy; GianLuca.RampioniVinciguerra@osumc.edu (G.L.R.V.); fcitron@mdanderson.org (F.C.); 4 Pathology Unit, Istituto Nazionale Tumori IRCCS, Fondazione G. Pascale, 80131 Napoli, Italy; r.dececio@istitutotumori.na.it (R.D.C.); n.losito@istitutotumori.na.it (N.S.L.); giosco80@gmail.com (G.S.); m.delsesto@istitutotumori.na.it (M.D.S.); 5 Clinical Trials Unit, Istituto Nazionale Tumori IRCCS, Fondazione G. Pascale, 80131 Napoli, Italy; l.arenare@istitutotumori.na.it (L.A.); piera.gargiulo@istitutotumori.na.it (P.G.); c.schettino@istitutotumori.na.it (C.S.); m.piccirillo@istitutotumori.na.it (M.C.P.); f.perrone@istitutotumori.na.it (F.P.); 6 Department of Mental Health and Public Medicine, Section of Statistics, Università degli Studi della Campania Luigi Vanvitelli, 80138 Napoli, Italy; simona.signoriello@unicampania.it (S.S.); vittorio.simeon@unicampania.it (V.S.); paolo.chiodini@unicampania.it (P.C.); 7 Istituto di Ginecologia e Ostetricia, Università Cattolica del Sacro Cuore, 00168 Rome, Italy; 8 Urogynaecological Medical Oncology, Istituto Nazionale Tumori IRCCS, Fondazione G. Pascale, 80131 Napoli, Italy; s.cecere@istitutotumori.na.it (S.C.C.); s.pignata@istitutotumori.na.it (S.P.); 9 Pathology Unit, Department of Mental and Physical Health and Preventive Medicine, Università degli Studi della Campania Luigi Vanvitelli, 80138 Naples, Italy; montella.marco19@gmail.com; 10 Gynecologic Cancer Program, Università degli Studi di Milano, 20126 Bicocca, Italy; nicoletta.colombo@ieo.it; 11 Division of Obstetrics and Gynecology, ASST Spedali Civili di Brescia, 25123 Brescia, Italy; germanatognon@gmail.com (G.T.); bignottieliana@gmail.com (E.B.); 12 Pathology Unit, IRCCS CRO Aviano, National Cancer Institute, 33081 Aviano, Italy; vcanzonieri@cro.it

**Keywords:** ovarian cancer, Bevacizumab treatment, angiogenesis, microRNAs, vessel density

## Abstract

**Simple Summary:**

The possibility to identify, with appropriate biomarkers, patients that might mostly benefit from any given treatment is the basis of personalized oncology. Cancer biomarkers should be properly identified and validated on a large number of patients possibly enrolled in dedicated clinical trials. Here, we report the first molecular results of the MITO16A-ManGo-OV2 phase IV trial that was specifically designed to identify prognostic biomarkers of survival in epithelial ovarian cancer patients treated in first line with carboplatin-paclitaxel plus Bevacizumab (NCT01706120), a treatment for which validated predictive or prognostic biomarkers are still lacking. With this work we propose not only novel possible biomarkers for Bevacizumab-treated patients but also a way through which they can be properly collected, analyzed and statistically evaluated in the frame of large multicenter clinical trials.

**Abstract:**

Background. Epithelial ovarian cancer (EOC) is a rare, highly lethal disease. In a subset of high grade EOC patients, maintenance therapy with the antiangiogenic drug Bevacizumab (BEV) is a valuable option. To date, no validated predictive or prognostic biomarkers exist for selecting EOC patients that might benefit from BEV treatment. Methods. Immunohistochemistry and RT-qPCR evaluated the expression of seven angiogenesis-related proteins and of a twelve microRNAs angio-signature in EOC patients, treated in first line with chemotherapy plus BEV (MITO16A/ManGO OV-2 phase IV trial). Centralized statistical analyses assessed the associations between each biomarker, clinical prognostic factors and survival outcomes. Results. High miR-484 expression was associated with longer progression-free and overall survival. Notably, the combined expression of miR-484 and its target VEGFB identified a subset of patients that might mostly benefit from BEV treatment. No other significant correlations were found between the other analyzed biomarkers and patients’ survival. The application of a shrinkage procedure to adjust for over-fitting hazard ratio estimates reduced the association significance. Conclusions. The analysis of angiogenesis related biomarkers in EOC patients homogenously treated with BEV in first line provides novel insight in their prognostic value and suggests that some of them might merit to be tested as predictive markers of drug activity in dedicated randomized trials.

## 1. Introduction

Epithelial ovarian cancer (EOC) is a rare but highly lethal disease (2.5% of all cancers in women; 46% survival at 5 years). At least four morphologically and molecularly distinct histotypes exist, i.e., high and low grade serous, endometrioid and clear cell carcinomas. However, EOC patients are currently all treated with first line platinum (PT)-based chemotherapy. Response to PT is highly predictive of patients’ prognosis and dictates the choice of subsequent lines of treatment [1,2].

For high grade (HG) EOC, maintenance strategies include the use of Bevacizumab (BEV, anti-VEGFA antibody), both in first line and in recurrent PT-sensitive (PT-Sens) EOC patients. PARP inhibitors (PARPi) in Italy can be used in patients with BRCA1/2 mutated, HRD (homologous recombination deficiency) and even in homologous recombination proficient (HRP) tumors in first line and in a PT-Sens recurrent setting. Several clinical studies suggest that adding targeted agents (i.e., BEV and PARPi) to PT-based therapies improves the efficacy of PT + Taxane (TAX) first line therapy [3,4,5,6,7,8,9,10,11].

We recently proved that continuing BEV beyond progression combined with chemotherapy in patients with PT-Sens recurrent ovarian cancer improves progression-free survival compared with standard chemotherapy alone [12].

Since there is convincing clinical evidence regarding effectiveness of BEV in maintenance setting, there is an urgent need to identify responders who would benefit from this intervention via biomarkers. Other patients may derive more from other options (e.g., PARPi). 

To this aim, in 2012, we designed the MITO16A-ManGO OV-2 phase IV trial that explored if selected clinical and biological factors could identify patients with better prognosis in terms of progression free survival (PFS) and overall survival (OS) after combined treatment with chemotherapy and BEV [13].

Here we report the prognostic role of selected angiogenesis-related biomarkers in the MITO16A-ManGO OV-2 phase IV trial population. In particular the prognostic significance of microvessel density (MVD) was evaluated using the CD31 that the previous report associated to the response to BEV [14]. To distinguish more mature vessels [15,16], CD31 plus Alfa-Smooth Muscle Actin (α-SMA) double staining was used. The expression of vascular endothelial growth factor A and B (VEGFA and VEGFB) and their receptors (VEGFR1 and VEGFR2) was tested to verify if VEGF pathway modification could also have a prognostic role, as proposed [17,18,19]. Finally, based on our observation that the expression of a specific microRNA defines chemoresistance in EOC through modulation of angiogenic factor, we also evaluated the expression and prognostic significance of miR-484 that, in ovarian cancer, modulate the expression of both VEGFB in cancer cells and, upon secretion, of VEGFR2 in endothelial cells [20]. 

## 2. Materials and Methods

MITO16A-ManGO OV-2 is a phase IV registered trial (EudraCT number: 2012-003043-29, Registered 24 September 2012, (www.clinicaltrials.gov number: NCT01706120)) that aims to explore the prognostic role of selected biomarkers in EOC patients treated in first line with chemotherapy (Paclitaxel + Carboplatin × 6) plus BEV (15 mg/kg) for 15 months [13]. The full protocol is provided as Supplementary Information attached to this manuscript. All patients provided written informed consent. Twelve research groups designed the trial as an exploratory study and no a priori hypothesis was defined to calculate the sample size of the trial. Here we reported the analyses related to angiogenic biomarkers performed at Istituto Nazionale dei Tumori IRCCS—Fondazione G. Pascale (VEGFA and Hypoxia-Inducible Factor 1-alpha, (HIF1-α)), at the Fondazione Policlinico Universitario A. Gemelli IRCCS (CD31 and α-SMA) and at the Centro di Riferimento Oncologico di Aviano (CRO), IRCCS, National Cancer Institute (VEGFR1, VEGFR2, VEGFB and microRNAs expression). Other evaluated biomarkers, not strictly related to angiogenesis, will be reported in future dedicated studies. With a sample size of 400 patients and after the registration of 280 events (either for PFS or OS), the study was designed to have 80% power to identify a prognostic factor able to select a favorable subgroup with a 0.60 HR, for a presumed expression of the favorable prognostic factors in 20% of the population, with alpha level of 0.01. 

**Figure 1:**
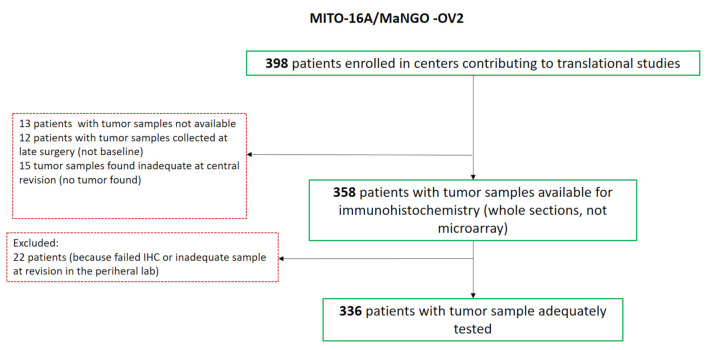
Consort of the study.

A total of 398 patients were enrolled in the study and tissue sample collection was centralized at the INT G. Pascale of Naples that supervised the quality controls and performed tissue processing to build TMA, to extract nucleic acid and provide final investigator of tissue slides as necessary. The utilized procedures were recently reported elsewhere [21]. The consort of analyzed samples is provided in Figure 1.

### 2.1. Tissue Micro Array (TMA) Building for IHC Analysis

TMAs were built taking the most representative areas from each single case. The whole MITO16 TMA contains 358 tissue samples from primary (236) and metastatic (122) ovarian cancer samples distributed in 7 TMA, together with internal controls (2 Fallopian tube samples, IGROV1 and SKOV3 cells). Three 1 mm cores were collected from each eligible tumor block and arrayed into a recipient paraffin block (35 × 20 mm) using a tissue array instrument (Galileo CK3500 TMA, ISENET, Milan, Italy) as described [21].

### 2.2. RNA Extraction, Quality Controls and microRNA Amplification

Total RNA was extracted from two 1 mm cores of FFPE tissues using the QIAGEN miRNeasy FFPE Kit as described [21]. RNA concentration was assessed by the NanoDrop 2000 UV-Vis spectrophotometer and samples with RNA concentration lower than 80 ng/µL were excluded from the study. Sample purity and RNA integrity was evaluated as described [21]. 

Total RNA was retro-transcribed and converted in cDNA using TaqMan-based technology and the TaqMan Reverse Transcription Kit (Applied Biosystems, Paisley, UK). Twelve microRNAs were amplified by qRT-PCR using specific TaqMan probes with the TaqMan Master Mix reagent (Applied Biosystems) in 384 well plates (CFX384 qRT-PCR Thermocycler, Bio-Rad, Milano, Italy). Based on our previous results [20], specific probes (Thermo Fisher Scientific, Paisley, UK) were used to amplify miRs hsa-miR-484 (#001821), hsa-miR-19a (#000395), hsa-miR-483 (#002338), hsa-miR-181a (#000480), hsa-miR-491 (#001038), hsa-miR-744 (#002324), hsa-miR-671 (#002322), hsa-miR-642 (#001592), hsa-miR-592 (#001546), hsa-miR-653 (#002292), hsa-miR-217 (#002337), hsa-miR-302d (#000535) in 328 RNA samples starting from 100ng RNA for RT reactions. Each sample has been amplified in duplicate in two independent replicates. Normalized miRs expression (considering U6, #001973 as reference), calculated using the comparative Ct method, was used for statistical analyses. As pre-specified condition, tumor samples were considered negative for the expression of miRs when a mean Ct > 34 cycles was observed. 

### 2.3. Immunohistochemistry Analyses 

Histological sections (5 μm) were made from the paraffin blocks or TMAs. Whole tissue sections were used to evaluate CD31 and α-SMA expression, while TMAs were used for the other biomarkers.

A double immunostaining procedure on whole sections was performed with CD31 (JC70A clone, Agilent Technologies, Santa Clara, CA, USA) and α-SMA (EPR5368 clone, Abcam, Cambridge, UK). Deparaffinization, rehydration and epitope retrieval of tissue specimens were performed with pH 6-Target Retrieval Solution in DAKO PT Link module (Agilent Technologies). After blocking the endogenous peroxidase activity, the slides were incubated with 20% normal goat serum for 30 min at room temperature and then with a combination of mouse anti-CD31 (1:50) and rabbit anti-α-SMA (1:2500) primary antibodies for 30 min at RT. For double staining IHC, ImmPRESS Duet Staining HRP/AP Polymer Kits (Vector Labs, Peterborough, UK) was used according to the manufacturer’s instructions. The immunoreaction for CD31 was detected by anti–mouse IgG-HRP antibody (brown staining) while the α-SMA immunoreaction was detected with the alkaline phosphatase substrate (magenta staining). Sections were counterstained with hematoxylin, dehydrated and mounted. Staining without primary antibody was used as a negative control.

Tumor sections stained with CD31 and α-SMA were examined at low magnification (×20) to identify areas containing highest density of microvessels (MVD) (hotspots). For each hotspot, CD31- and α-SMA/CD31-positive (pericyte-covered vessels) intratumoral microvessels were counted blindly under a microscope field (×400 objective magnification, high-power field area = 0.237 mm^2^). Tumor MVD was evaluated by averaging the number of CD31+ vessels from at least three distinct areas of the tumor and data expressed as number of vessels per mm^2^. Pericytes were defined as a single layer of α-SMA-positive cells co-localized with CD31+ microvessels. Finally, the fraction of mature vessels per mm^2^ was calculated as α-SMA+MVD/MVD ratio. 

TMAs sections were deparaffinated with xylene according to standard procedures, followed by rehydration through serial ethanol treatments. For VEGFR1 and VEGFR2 slides, antigen retrieval enhancement was performed in citrate buffer (0.01 M sodium citrate (pH 6.0)) and heated in a microwave oven at 600 W (three times for 5 min each). For VEGFB, antigen retrieval was performed using Target Retrieval Solution High pH 8 (cat. #DM812, Dako, Santa Clara, CA, USA) and heated in a microwave oven at 270 W (three times for 5 min each). Each slide was incubated for 1 h at room temperature with blocking solution (PBS 0.1% Tween and 5% normal goat serum). Staining was performed incubating overnight at 4 °C the listed antibodies: VEGFR1 (dilution 1:200, clone CL0344, cat. #AMAB90703, Sigma-Aldrich, Milano, Italia), VEGFR2 (dilution 1:500, clone D5B1, cat. #9698, Cell Signaling, Beverly, MA, USA), VEGFB (dilution 1:100, clone OTI1H9, cat. #MA5-26326, Thermo Fisher Scientific), followed by DAB reaction (cat. #SK-4100, Vector, Lab. Burlingame, CA, USA), according to manufacturer’s protocol and standard procedures.

For VEGFB, VEGFR1 and VEGFR2 IHC, staining was scored according to intensity of staining (0 = negative, +1 = weak positivity, +2 = moderate positivity, +3 = strong positivity). Samples scored as 0/+1 or +2/+3 were considered negative or positive, respectively. In positive tumors, the percentage of stained cells was further evaluated.

TMA sections were stained with HIF1-α and VEGFA antibodies using a Leica Bond-III autostainer. Visualization of the antibody–antigen reaction was performed using a Bond polymer refine detection kit (S9800). For VEGFA staining, antigen retrieval was performed at pH 6.0 and the anti-VEGFA antibody (clone VG1, Dako) used at the dilution of 0.6 µg/mL in Dako AR9352 diluent solution. For HIF1-α staining antigen retrieval was performed at pH 8.0 and the anti- HIF1-α antibody (clone 67, Santa Cruz Biotechnology, Heidelberg, Germany) used at the dilution of 40 µg/mL in Dako AR9352 diluent solution. In each TMA core, we estimated the percentages and the expression intensities of HIF1-α and VEGFA scoring as positive all samples with cytoplasmic staining (+1 weak positive intensity, +2 strong positive intensity).

### 2.4. Statistical Analyses 

Continuous variables were described with median values and interquartile range (IQR), qualitative variables were expressed in terms of absolute numbers and relative frequency.

For all biomarkers analyzed, a histogram was used to describe the distribution and to check the presence of high frequencies of 0 values.

A scatterplot and a modified version of Kendall test for zero-inflated values were used to test the correlation between biomarkers [22].

The associations between biomarkers and the clinical prognostic factors were investigated using the Wilcoxon rank test for zero-inflated data (ZIW) for dichotomous variables and the Kruskal–Wallis zero inflated (ZIKW) for categorical variables using a permutation test. The prognostic effect of each biomarker was evaluated using progression free survival (PFS) and overall survival (OS) as endpoints. PFS was defined as the time elapsing from the inclusion into the study to the first occurrence of either death for any cause or disease progression. OS was defined as the time elapsing from the inclusion into the study and death for any cause.

Kaplan–Meier curves were drawn for PFS and OS and compared with a two-sided log-rank test.

To test the prognostic role for each biomarker on both PFS and OS univariable and multivariable, Cox proportional models were performed.

In a first univariable analysis, the biomarker was tested as continuous variable after testing the linearity assumption using fractional polynomial and a dummy variable to estimate effect of 0 value. In a second univariable analysis, the biomarker was tested as a categorical variable after searching for the best cutoff value that minimizes the *p*-value of hazard ratio (HR). The best cutoff was selected among the biomarker values choosing the value that minimized the *p*-value of hazard ratio (HR) for the categorical variable defined by the cutoff value. The best cutoff search was calculated on PFS and then applied to the OS. 

For each biomarker (and for both continuous effect and best cutoff categories), a multivariable analysis was performed using as covariates: age (as category <65 vs. ≥65), ECOG performance status (PS) (0 vs. 1–2), residual disease (None; ≤1 cm; >1 cm; not operated), FIGO stage (III vs. IV) and tumor histology (high grade serous vs. other). Covariates were chosen according to the model defined in the manuscript reporting the clinical results of this trial [13]. A shrinkage procedure with 95% CI was calculated with bootstrap-percentile method [23] to adjust for over-fitting HRs estimates of best cutoff categories. Data were analyzed using R software version 3.6.0 (R Foundation for Statistical Computing, Vienna, Austria) Prism 8.2.0 (GraphPad Software Inc., San Diego, CA, USA) and STATA/MP 14.1 (StataCorp LP, College Station, TX, USA).

## 3. Results

A total of 398 patients were enrolled in the study and agreed to donate their samples for translational studies. Forty-two patients were excluded for different reasons (Figure 1). Samples from 356 patients were sent to peripheral research centers for IHC and microRNA (hereafter defined miR) expression analyses. Twenty patients were excluded at this stage for technical reasons (Figure 1).

No differences in clinical and pathological variables were observed between patients included in biomarkers analyses (*n* = 336) and the whole MITO16A trial population (*n* = 398) (Table 1).

### 3.1. Immunohistochemistry Evaluation of Tumor Vasculature and VEGF Family Members

**Figure 2:**
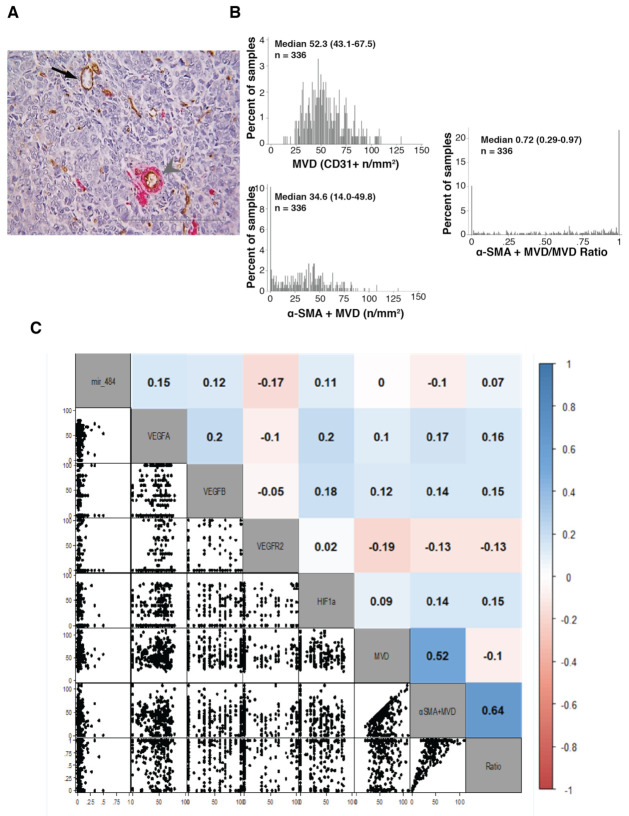
Evaluation of MVD in MITO16A population and its correlation with selected angiogenesis-related genes. (**A**). Representative picture showing CD31-positive intratumoral microvessels (arrow) and CD31-positive entities associated with α-SMA positive cells (arrowhead) (magnification 40×, scale bar 100 μm). The microvessel density (MVD) and the αSMA+ microvessel density (αSMA + MVD) were expressed as mean number of vessel per mm^2^. (**B**). Distribution of CD31 and α-SMA expression in the analyzed samples. On the left, the distribution of α-SMA + MVD/MVD ratio is shown. (**C**). Pairwise distribution of biomarkers. The correlogram plot reports the Kendall’s tau values. On the right is shown the color scale used.

Microvessel density (MVD) was evaluated using CD31 on entire tissue slides from 336 patients. α-SMA was evaluated to quantify pericyte coverage of endothelial vessels on the same slides as a measure of more mature vessels [15,16]. Median MVD in the analyzed samples was 52.3 with 43.1–67.5 interquartile range (IQR) (Figure 2A,B). 

The median MVD of α-SMA positive CD31 positive vessels was 34.6 (IQR 14–49.8) with a median ratio between α-SMA + MVD and MVD of 0.72, indicating that most but not all CD31 positive vessels were covered by α-SMA-positive pericytes. Accordingly, high correlation (0.64, range 0.59–0.70) was observed between α-SMA-MVD and α-SMA-MVD/MVD ratio using the Kendall correlation test (Figure 2C).

We then evaluated if VEGF family members’ expression correlated with the extent of MVD in primary EOC, looking at the expression of VEGFA and VEGFB growth factors and their receptors VEGFR1 and VEGFR2. To this aim we used TMA tissue slides which carried three core section of each of 358 available samples corresponding to 313 patients [21]. The use of TMA forced us to examine and quantify the expression of the chosen biomarkers only in tumor cells. All but two cases were negative for VEGFR1 expression on tumor cells and therefore this biomarker was excluded from the analyses. Due to different technical reasons encountered by the different laboratories, we could analyze the expression of VEGFA on 353 cases belonging to 306 patients and of VEGFB and VEGFR2 on 337 and 324 cases belonging to 296 and 284 patients, respectively (Supplementary Figure S1). 

VEGFA was expressed in the vast majority and VEGFB in about 65% of analyzed samples (Supplementary Figures S2 and S3). No differences were noted among paired samples derived from the ovary (primary tumor) and distant peritoneal localizations (metastasis) in term of VEGFA/B positivity (Supplementary Figures S2 and S3). Accordingly, in paired samples from the same patients a good correlation was noted for both VEGFA and B (Supplementary Figures S2E and S3D). Only a minority of samples expressed VEGFR2 on tumor cells in both primary and metastatic localization with a good correlation between different lesions in paired samples (Supplementary Figure S4). We did not find any significant correlation between VEGF family members and tumor MVD (Figure 2C).

### 3.2. Evaluation of VEGF Family Member Regulators Expression

Based on our previous results and the current literature, we next tested if known regulators of VEGFA/B expression, namely, HIF1-α and a microRNA signature, identified in platinum-resistant ovarian cancers [20,24], correlated with the expression of VEGF family members and/or with the extent of intratumor MVD.

HIF1-α, analyzed on 302 patients (Supplementary Figure S1D), was expressed in about 60% of samples with a slightly higher expression in metastatic lesions with respect to primary tumors, although a significant correlation was noticed between primary and metastatic localization in paired samples from the same patients (Supplementary Figure S5). HIF1α expression did not correlate with either VEGFA/B or MVD, as previously reported for a small cohort (*n* = 60) of ovarian cancer cases [25].

Next, we tested the expression of an angiogenesis-related microRNA signature composed of 12 microRNAs (miRs) whose expression correlated with the response to platinum and the extent of tumor vascularization in ovarian cancers [20]. Seven miRs had measurable levels expressed in at least 90% and, among them, four in 100% of tested samples (Supplementary Figure S6A). Here we focused on miR-484 for its reported role of possible regulator of angiogenesis in ovarian cancer by controlling VEGFB and VEGFR1 expression (Supplementary Figure S6B and [20]). Normalized miR-484 expression was similar between primary and metastatic localizations with a good, but not highly significant, correlation in paired samples from the same patients (Supplementary Figure S6C–E). A subset of patients (17/47) showed increased expression of miR-484 expression in metastatic lesions (Supplementary Figure S6F).

Yet, Kendall correlation test did not confirm the previously observed association of miR-484 with MVD evaluated by CD31 expression and/or αSMA-CD31 expression or ratio, although there was a tendency toward negative correlation (i.e., −0.10) when αSMA-positive vessels were considered (Figure 2C). 

### 3.3. Coupling Biomarkers Expression with Clinical Variables and Patients’ Outcome

We tested the association between the expression of the evaluated biomarkers with known prognostic factors (age, tumor histology, FIGO stage, performance status (PS), residual disease and baseline hypertension). The only significant associations were found for age with α-SMA + MVD/MVD ratio and for PS with VEGFB (Figure S7A,B). When analyzed as continuous variables in a univariable or multivariable models, no biomarker was able to predict a patient’s prognosis for PFS and OS (Supplementary Table S1). These data prompted us to identify for each biomarker the best cutoff that minimizes the *p*-value of hazard ratio (HR) (see methods for details). Using these cutoff values, we tested the abilities of the different biomarkers to predict the prognosis of patients using Kaplan–Meier curves both in PFS and OS (Supplementary Figures S8 and S9). In univariable analyses, before applying shrinkage procedure, only high miR-484 expression predicted longer patients’ survival both in terms of PFS (HR = 0.72, *p* = 0.023) and, notably, OS (HR = 0.61; *p* = 0.016) (Supplementary Figures S8 and S9 and Table S2). High MVD was slightly associated with PFS (HR = 0.65; *p* = 0.043) (Supplementary Figure S8 and Table S2). However, these significant associations disappeared after HR adjustment for over-fitting (Table S2).

We next tested the predictive value of the same biomarkers in multivariable analyses adjusted for patients’ clinical characteristics. Again, only high miR-484 expression was associated with OS (HR = 0.59; *p* = 0.012) but not PFS (Table 2). Moreover, in this case, after the application of a shrinkage procedure to adjust for over-fitting HRs estimates, miR-484 expression lost its statistical significance (Table 2). 

### 3.4. Testing the Potential of Combining Biomarkers to Predict Patients’ Outcome

**Figure 3:**
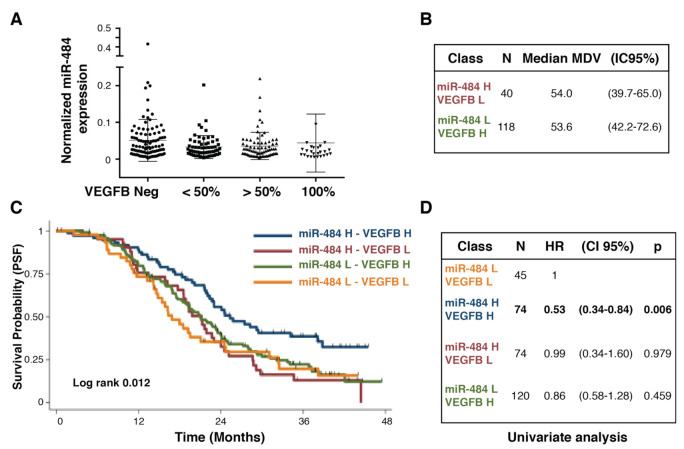
Evaluation of the prognostic role of miR-484/VEGFB combined expression. (**A**). Dot plot reporting the expression of miR-484 in EOC samples divided based on the expression of VEGFB (Neg = negative samples; <50% samples expressing VEGFB in less than 50% of the cells; >50% samples expressing VEGFB in 50–90% of the cells. 100% samples with all (i.e., 90–100%) cells expressing VEGFB. (**B**). Evaluation of MVD (microvessel density) in samples expressing high levels of miR-484 (miR-484 H) and low levels of VEGFB (VEGFB L) or expressing low levels of miR-484 (miR-484 L) and high levels of VEGFB (VEGFB H) (**C**). Kaplan–Meier analysis evaluating the progression free survival of MITO16A-MANGO OV2 patients, divided based on the expression of VEGFB and miR-484. (**D**). Table reporting the univariate analysis of the different group analyzed in C. HR = hazard ratio. CI = confidence interval.

Finally, based on our previous results showing that miR-484 targets VEGFB in ovarian cancer cells [20], we closer examined their behavior in the studied population. We observed that miR-484 expression was higher in VEGFB negative samples and lower in samples 100% positive for VEGFB (Figure 3A, p between the two groups = 0.017). This observation prompted us to investigate if the two groups (i.e., high miR-484 and low VEGFB expression vs. low miR-484 and high VEGFB expression) had different MVD, but this was not the case (Figure 3B). Of note, high miR-484 combined with high VEGFB expression significantly predicted longer PFS (Figure 3C,D).

The figure was generated using the Illustrator software to combine files generated in PRISM and R software.

## 4. Discussion

Here we report the first molecular results of the MITO16A-ManGo-OV2 phase IV trial that was specifically designed to identify prognostic biomarkers of survival in EOC patients treated in first line with carboplatin-paclitaxel plus BEV schedule, followed by BEV maintenance. In the treated high risk population (Stage IIIb-IV EOC), we observed a median PFS time of 20.8 months with a 32.3 month follow up period [13], similar with the one originally observed in the ICON7 and GOG-218 trials with a 36 month follow up [3,4], supporting the evidence that BEV improves PFS of EOC patients when used as first line treatment. 

We were able to analyze 84.4% of the intention to treat population for MVD and completed the analysis with the use of α-SMA as a measure of more mature vessels. Both CD31 and α-SMA/CD31 staining were not able to predict survival in BEV treated patients. Our results are in line with the ones reported by the GOG-218 investigators who recently showed no prognostic or predictive value for CD31 expression among the 1,438 patients included in the final overall survival analysis of the study [26,27]. These results confirm that basal tumor MVD cannot be considered a good prognostic marker for BEV treated EOC patients and suggest that the evaluation of α-SMA expression does not add significant prognostic value. Yet, it would be interesting verifying if CD31, and/or other variables considered here, could predict survival in the control group of the randomized MITO16B-ManGo-OV2 phase III trial testing the efficacy of BEV retreatment to prolong PFS beyond progression basal [12]. 

We also confirmed the previous evidence suggesting that tumoral expression of VEGFA and VEGFR2 were not able to predict the prognosis in BEV treated patients. In our cohort, we observed high expression of VEGFA in about the half of the 306 tested patients, but the protein levels were not prognostic in these patients. However, it should be noted that VEGFA could be expressed also as an antiangiogenic alternative spliced form (VEGFAb) that is not distinguishable from the proangiogenic (VEGFAa) growth factor by the available antibodies [28]. It would be interesting to test if the VEGFA mRNA levels and, especially, the expression of VEGFAb antiangiogenic spliced forms, could conversely be associated with the response to BEV as recently observed in colon cancer and proposed for ovarian cancer [29,30]. 

We tested the potential prognostic role of miR-484 and of its target VEGFB, previously associated with higher platinum sensitivity in high grade ovarian cancer [20]. We found that high miR-484 expression was associated with longer survival of the tested population especially when overall survival was considered, supporting the possibility that high miR-484 expression defines a platinum sensitive population, as originally proposed [20]. However, much more research is needed to confirm this possibility. 

From a methodological point of view, we want to highlight that the application of a shrinkage procedure, aimed to adjust for over-fitting HRs estimates, failed to confirm the prognostic value of miR-484. To the best of our knowledge, this statistical correction is not very often used to validate the prognostic/predictive value of candidate biomarkers in oncology. We thus propose that, in the future, more stringent statistical plans should be required to claim the role of biomarkers in oncology. 

In the present analyses, we cannot confirm the correlation between miR-484 expression and MVD previously observed in preclinical models and in a subset of analyzed patients [20]. This discrepancy could be related to different aspects including the use of a different technology to evaluate miRs expression and/or CD31 or a different method to quantify the MVD. 

More intriguing are the results showing no inverse correlation between VEGFB and miR-484 expression in the analyzed samples based on the knowledge that VEGFB is a bona fide miR-484 target in ovarian cancer cells [20]. These data might merit some deeper discussion. First, to meet the needs to perform multiple biomarkers analyses on a large population we decided to limit the analyses on whole tissue sections to the ones absolutely necessary, namely, MVD markers (this manuscript), tumor lymphocytes infiltration and IL6 and pSMAD2 expression (manuscripts in preparation). All other planned biomarkers were evaluated on TMA sections. Moreover, RNA was extracted starting from core biopsies of the tissue samples [21] and not from the entire section. These approaches could have had an impact on the possible correlation between miR-484 and VEGFB. Moreover, we observed a good but not strong correlation in the expression of both miR-484 and VEGFB in paired primary and metastatic samples, suggesting that their expression could be modified by the local microenvironment that could impact on their expression correlation. However, we confirmed that in the extreme groups there was an inverse expression of miR-484 and VEGFB. In particular, we observed that in VEGFB negative samples, miR-484 levels were significantly higher when compared to levels observed in VEGFB 100% positive samples. The presence of extreme groups had prognostic significance since high expression of miR-484 and VEGFB predicted longer PFS in BEV treated patients.

We are aware that this work has several limitations that should be taken into account. Due to technical reasons, the prespecified sample size of 400 patients for biomarker analysis has not been reached and this fact could have influenced the power of the study. Moreover, correction for multiplicity of testing and application of shrinkage procedure failed to confirm biomarker prognostic value and this should be taken into account in the design of future clinical trials. Some biomarkers evaluated on TMA on tumor cells (e.g., VEGFR1 and VEGFR2) could have had a prognostic role if their expression had been evaluated on stroma and endothelial cells. Finally, we want to highlight that the expression of HIF1-β, that functions as heterodimers in combination with HIF1-α and not evaluated here, could also have a prognostic significance in BEV treated patients.

## 5. Conclusions

Overall, we report the results of the first study specifically designed to identify prognostic biomarkers in patients treated in first line with BEV as maintenance therapy in EOC patients. Although we can say that the expression of the analyzed angiogenesis-related genes does not have clear prognostic significance, the study provided interesting evidence suggesting that some of the analyzed biomarker might instead have predictive significance of BEV activity. This is something that we would like to test using the samples collected in the frame of the MITO16B-ManGo-OV2 phase III trial. In these challenges, we think it would be worth using multiple biomarkers evaluated together as the combined evaluation of miR-484 and VEGFB seems to suggest.

## Figures and Tables

**Table 1:** Patient population in analysis.

Variables	Patients in Analysis (*n* = 336)		MITO16A Population (*n* = 398)	
**Age**, median (IQR)	59.3	(50.0; 66.5)	59.2	(49.9; 66.5)
**Age category**, *n*(%)				
<65	234	(69.6)	278	(70.0)
≥65	102	(30.4)	120	(30)
**ECOG performance status**, *n*(%)				
0	265	(78.9)	315	(79.2)
1–2	71	(22.1)	69	(17.3)
Missing			14	(3.5)
**Residual disease**, *n*(%)				
None	129	(38.4)	153	(38.4)
≤1 cm	68	(20.2)	72	(18.1)
>1 cm/	102	(30.4)	120	(30.2)
not operated	37	(11.0)	53	(13.3)
**FIGO stage**, *n*(%)				
IIIB	31	(9.2)	36	(9.1)
IIIC	233	(69.3)	275	(69.1)
IV	72	(21.4)	87	(21.9)
**Tumor histology**, *n*(%)				
High grade serous	290	(86.3)	333	(83.7)
Low grade serous	12	(3.6)	13	(3.3)
Endometrioid	9	(2.7)	9	(2.3)
Clear cell	10	(3.0)	11	(2.8)
Mucinous	1	(0.3)	3	(0.8)
Mixed	2	(0.6)	4	(1.0)
Other	12	(3.6)	25	(6.3)
**Baseline hypertension**, *n*(%)				
No hypertension	56	(16.6)	70	(17.6)
Prehypertension	176	(52.4)	199	(50.0)
On-AHT	104	(31.0)	122	(30.7)
Missing	0	-	7	(1.8)

Abbreviations. ECOG: Eastern Cooperative Oncology Group; FIGO: International Federation of Gynecology and Obstetrics; AHT: Arterial Hypertension Treatment.

**Table 2:** Multivariable analysis of biomarkers best cutoff for PFS and OS adjusted for clinical characteristics, original and shrunken coefficients.

**PFS**						
	**Original Coefficients**			**Shrunken Coefficient**		
	**HR**	**(95% CI)**	*p*	**HR**	**(95% CI)**	*p*
**MVD:**						
>31.2	0.74	(0.49–1.13)	0.165	0.87	(0.4–1.88)	0.715
**SMA_MVD:**						
>64.1	0.80	(0.53–1.22)	0.295	0.98	(0.44–2.19)	0.962
**Ratio:**						
>0.86	0.89	(0.68–1.17)	0.404	1.05	(0.42–2.61)	0.915
**MIR 484:**						
>0.017	0.71	(0.53–0.96)	0.023	0.76	(0.39–1.48)	0.421
**VEGFA:**						
>56.7	1.32	(0.99–1.75)	0.056	1.22	(0.63–2.38)	0.557
**VEGFB:**						
>45.0	0.69	(0.52–0.92)	0.011	0.73	(0.33–1.65)	0.453
**VGFR2:**						
>60.0	1.09	(0.75–1.60)	0.642	0.72	(0.17–2.99)	0.651
**HIF1-** **α** **:**						
>0.0	1.06	(0.79–1.40)	0.709	0.71	(0.23–2.19)	0.557
**OS**						
	**HR**	**(95% CI)**	*p*	**HR**	**(95% CI)**	*p*
**MVD:**						
>31.2	0.86	(0.48–1.53)	0.601	1.51	(0.18–12.91)	0.707
**SMA_MVD:**						
>64.1	0.74	(0.37–1.49)	0.406	1.14	(0.16–8.37)	0.897
**Ratio:**						
>0.86	0.86	(0.57–1.30)	0.484	1.16	(0.25–5.5)	0.848
**MIR 484:**						
>0.017	0.59	(0.39–0.89)	**0.012**	0.64	(0.20–2.08)	0.455
**VEGFA:**						
>56.7	0.85	(0.55–1.31)	0.451	1.14	(0.13–10.07)	0.909
**VEGFB:**						
>45.0	0.70	(0.46–1.06)	0.089	0.79	(0.18–3.52)	0.755
**VGFR2:**						
>60.0	1.19	(0.69–2.03)	0.531	0.77	(0.12–4.88)	0.779
**HIF1-** **α** **:**						
>0.0	1.24	(0.81–1.90)	0.332	0.99	(0.14–7.11)	0.990

Model adjusted for age (as category <65 vs. ≥65), ECOG performance status (0 vs. 1–2), residual disease (None; ≤1 cm; >1 cm; not operated), FIGO stage (III vs. IV) and tumor histology (high grade serous vs. other).

## Data Availability

No new data were created or analyzed in this study. Data sharing is not applicable to this article.

## Supplementary Materials

The following are available online at https://www.mdpi.com/article/10.3390/cancers13205152/s1, Protocol of study; Figure S1: Consort of analyzed biomarkers, Figure S2: Analyses of VEGFA expression in the MITO16A-MANGO OV2 trial, Figure S3: Analyses of VEGFB expression in the MITO16A-MANGO OV2 trial, Figure S4: Analyses of VEGFR2 expression in the MITO16A-MANGO OV2 trial, Figure S5: Analyses of HIF1-α expression in the MITO16A-MANGO OV2 trial, Figure S6: Analyses of microRNAs expression in the MITO16A-MANGO OV2 trial, Figure S7: Analyses of biomarkers expression with clinic-pathological variables of patients enrolled in the MITO16A-MANGO OV2 trial, Figure S8: Kaplan–Meier curves evaluating the progression free survival of patients enrolled in the MITO16A-MANGO OV2 trial, Figure S9: Kaplan–Meier curves evaluating the overall survival of patients enrolled in the MITO16A-MANGO OV2 trial, Table S1: Analysis of biomarkers (continuous and dummy for zero variables) adjusted for PFS and OS, Table S2: Analysis of biomarkers best cutoff for PFS and OS, original and shrunken coefficients.
