# Supplementary material for: Evaluation of Angiogenesis-Related Genes as Prognostic Biomarkers of Bevacizumab Treated Ovarian Cancer Patients: Results from the Phase IV MITO16A/ManGO OV-2 Translational Study
Source: Cancers (Basel). 2021 Oct 14;13(20):5152. doi: 10.3390/cancers13205152 (PMC8533892; doi:10.3390/cancers13205152)
Supplement: Supplementary file 1 [file cancers-13-05152-s001.zip › cancers-1405856-supplementary.pdf]

SUPPLEMENTARY INFORMATION

Evaluation of angiogenesis-related genes as prognostic biomarkers of Bevacizumab treated ovarian cancer patients: Results from the phase IV MITO16A/ManGO OV-2 translational study.

Daniela Califano, Daniela Gallo, Gian Luca Rampioni Vinciguerra, Rossella De Cecio, Laura Arenare, Simona Signoriello, Daniela Russo, Gabriella Ferrandina, Francesca Citron, Simona Losito, Piera Gargiulo, Vittorio Simeon, Giovanni Scambia, Sabrina Chiara Cecere, Marco Montella, Nicoletta Colombo, Germana Tognon, Eliana Bignotti, Gianfranco Zannoni, Vincenzo Canzonieri, Alessandra Ciucci, Anna Spina, Giosuè Scognamiglio, Michele Del Sesto, Clorinda Schettino, Maria Carmela Piccirillo, Franco Perrone, Paolo Chiodini, Sandro Pignata and Gustavo Baldassarre

.

CONTENTS:

Supplementary Figures S1 – S9 **Page 2 - 10**

Supplementary Table S1 - S2 **Page 11 - 12**

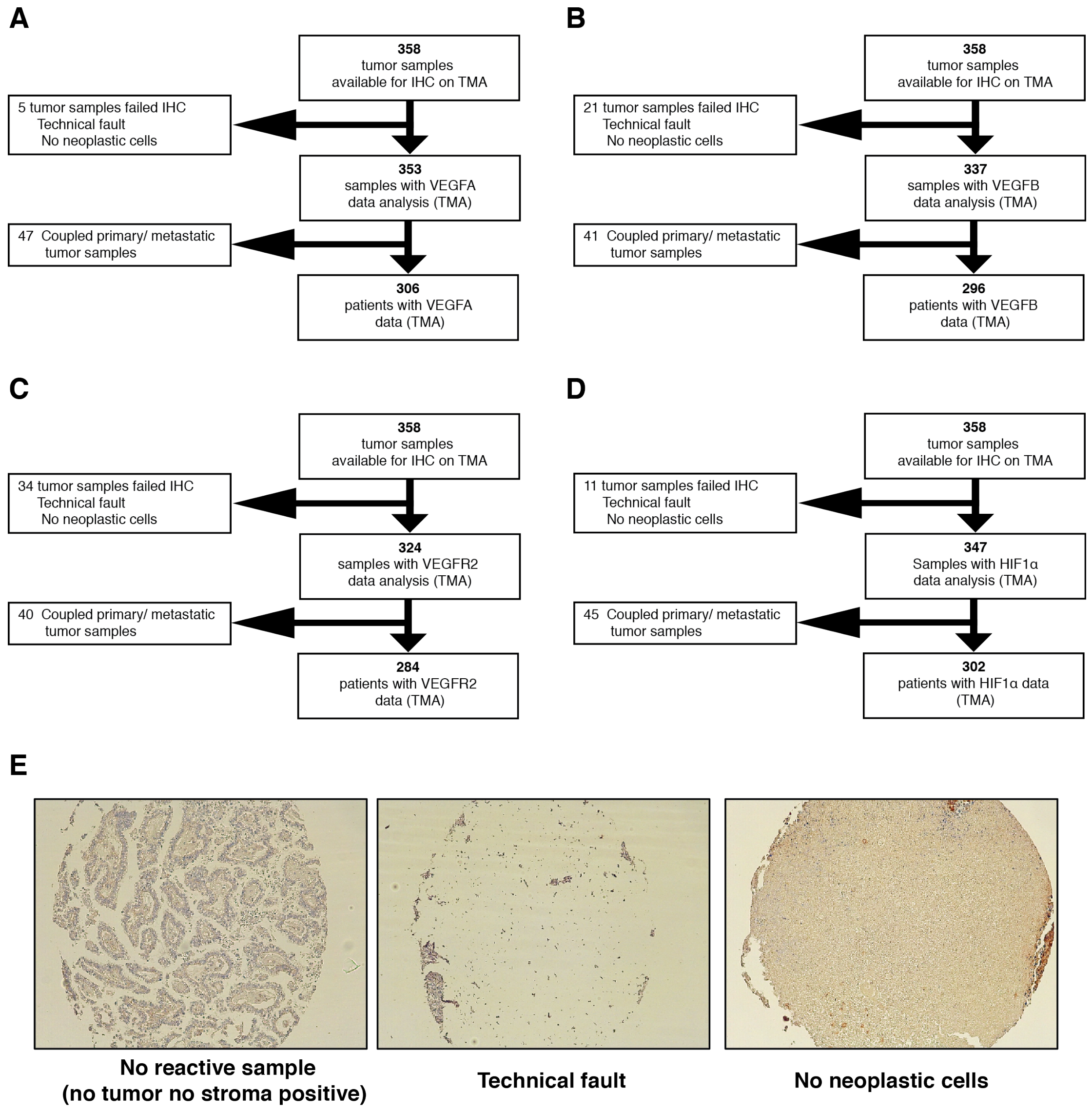

Figure S1. Consort of analyzed biormarkers.

A-D. Consort reporting the patients' samples analyzed for each of the indicated biomarkers and the causes of samples exclusions.

E. Typical examples of technical reasons leading to patients' exclusions

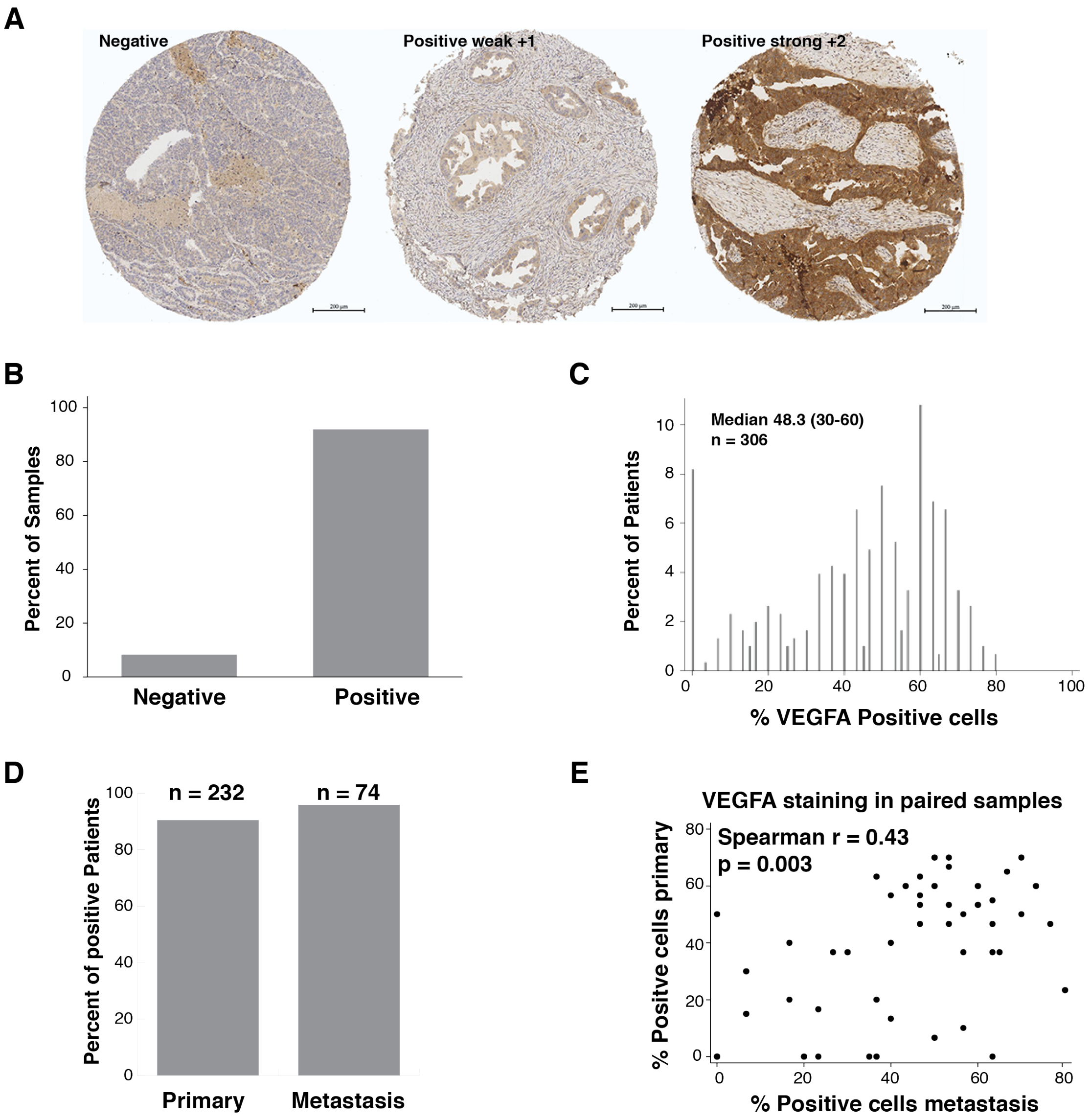

Figure S2. Analyses of VEGFA expression in the MITO16A-MANGO OV2 trial.

A. Typical examples of VEGFA expression in the analyzed Tissue Micro Arrays (TMA).

B. Graph reporting the percentage of negative and positive patients' samples in the analyzed TMA

C. Graph reporting distribution of analyzed samples based on the percentage of VEGFA positive cells in samples from each patient

D. Graph reporting the percentage of VEGFA positive samples among primary and metastatic lesion

E. Graph reporting the correlation of VEGFA expression in paired primary and metastatic lesions.

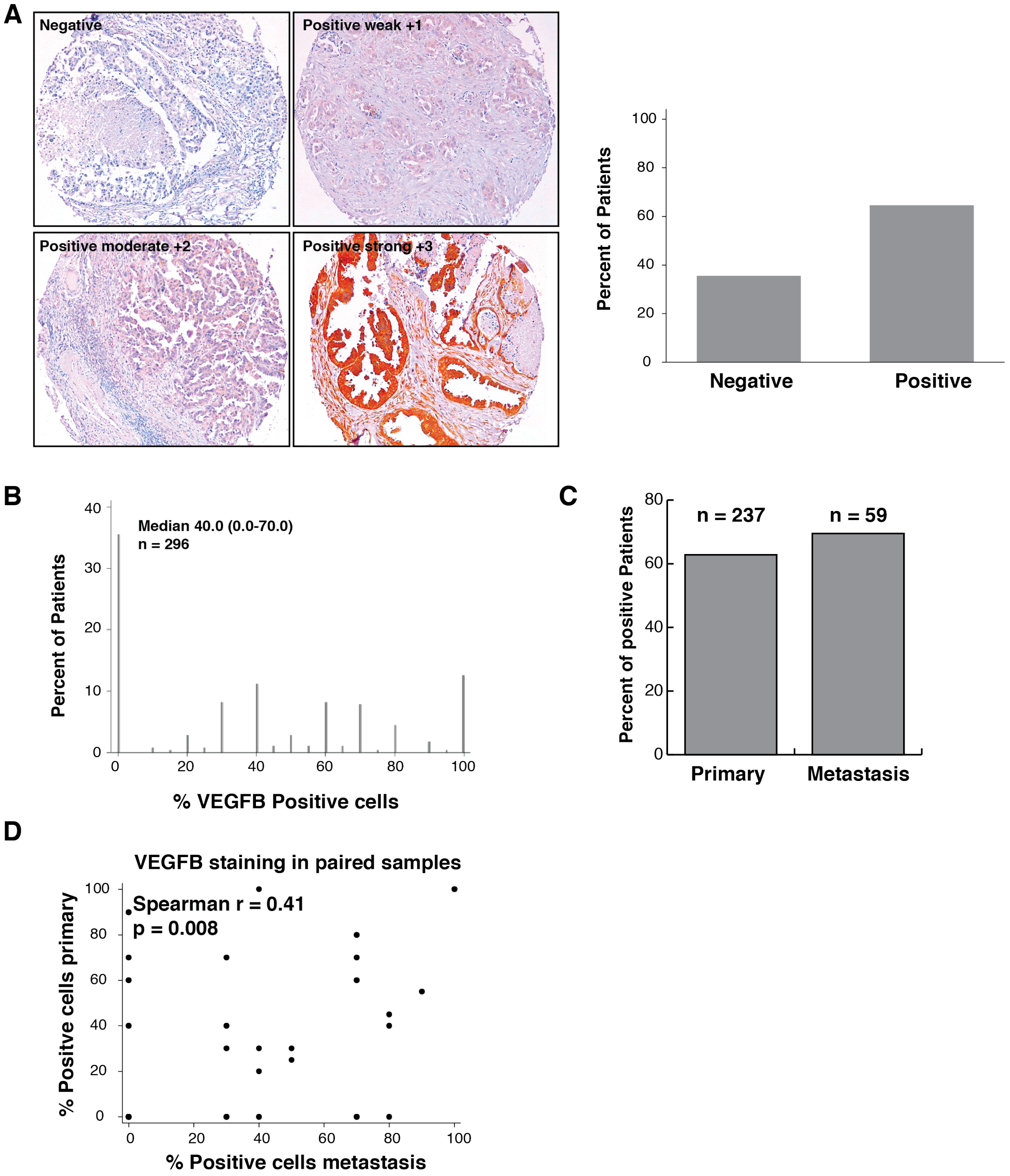

Figure S3. Analyses of VEGFB expression in the MITO16A-MANGO OV2 trial.

A. Typical examples of VEGFB expression in the analyzed Tissue Micro Arrays (TMA). On the right the graph reports the percentage of negative and positive patients' samples in the analyzed TMA

B. Graph reporting distribution of analyzed samples based on the percentage of VEGFB positive cells in samples from each patient

C. Graph reporting the percentage of VEGFB positive samples among primary and metastatic lesion

D. Graph reporting the correlation of VEGFB expression in paired primary and metastatic lesions.

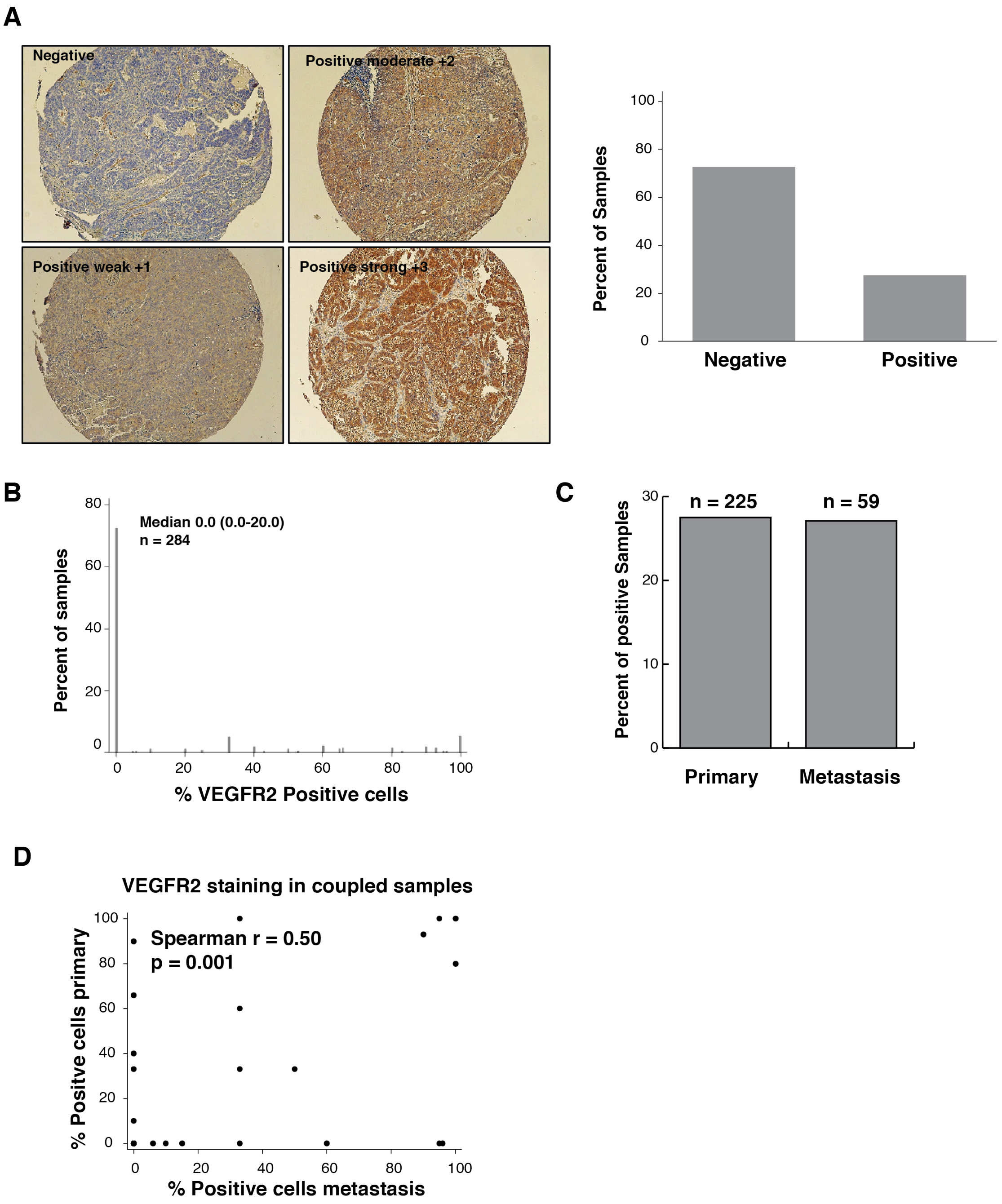

Figure S4. Analyses of VEGFR2 expression in the MITO16A-MANGO OV2 trial.

A. Typical examples of VEGFR2 expression in the analyzed Tissue Micro Arrays (TMA). On the right the graph reports the percentage of negative and positive patients' samples in the analyzed TMA

B. Graph reporting distribution of analyzed samples based on the percentage of VEGFR2 positive cells in samples from each patient.

C. Graph reporting the percentage of VEGFR2 positive samples among primary and metastatic lesion

D. Graph reporting the correlation of VEGFR2 expression in paired primary and metastatic lesions.

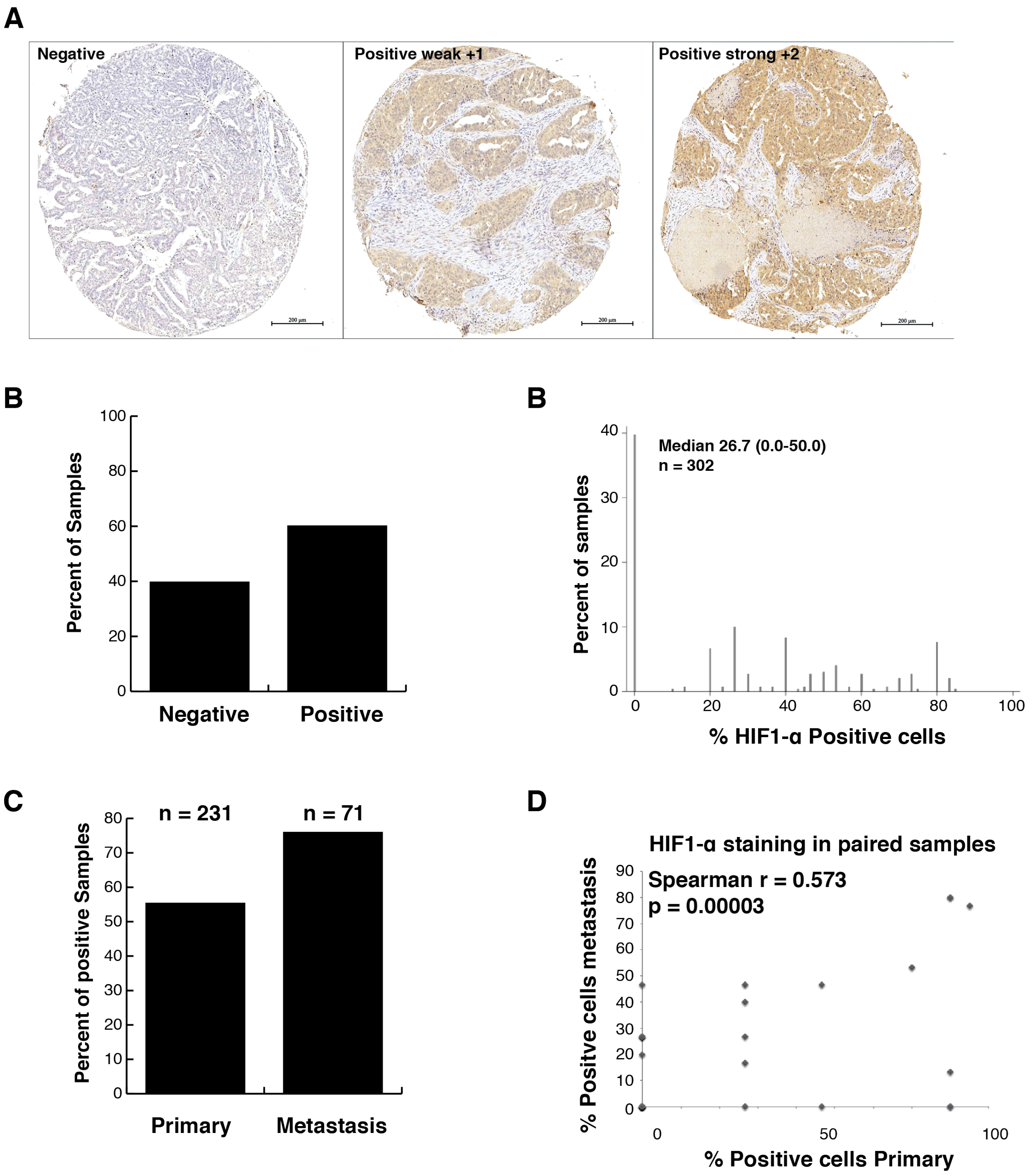

Figure S5. Analyses of HIF1-α expression in the MITO16A-MANGO OV2 trial.

A. Typical examples of HIF1-α expression in the analyzed Tissue Micro Arrays (TMA).

B. Graph reporting the percentage of HIF1-α negative and positive patients' samples in the analyzed TMA

C. Graph reporting distribution of analyzed samples based on the percentage of HIF1-α positive cells in samples from each patient.

D. Graph reporting the percentage of HIF1-α positive samples among primary and metastatic lesion

E. Graph reporting the correlation of HIF1-α expression in paired primary and metastatic lesions.

A

microRNA	Mean Expression	St. Dev.	Positive Samples
**hsa-miR-484**	**0,02543**	**0,0553**	**328/328**
hsa-miR-181a	0,03202	0,3068	328/328
hsa-miR-19a	0,01536	0,1199	328/328
hsa-miR-483-5p	0,00343	0,0455	327/328
hsa-miR-491	0,00073	0,0011	328/328
hsa-miR-744	0,00041	0,0005	310/328
hsa-miR-671	0,00022	0,0003	305/328
hsa-miR-642	0,00013	0,0003	140/328
hsa-miR-592	0,00010	0,0020	101/328
hsa-miR-653	N. A.	N. A.	0/328
hsa-miR-217	N. A.	N. A.	0/328
hsa-miR-302d	N. A.	N. A.	0/328

B

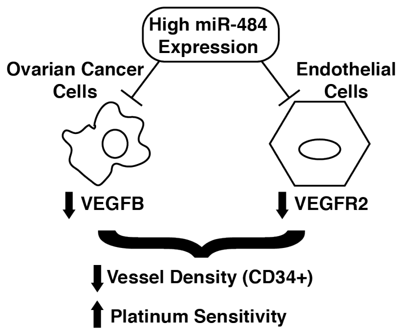

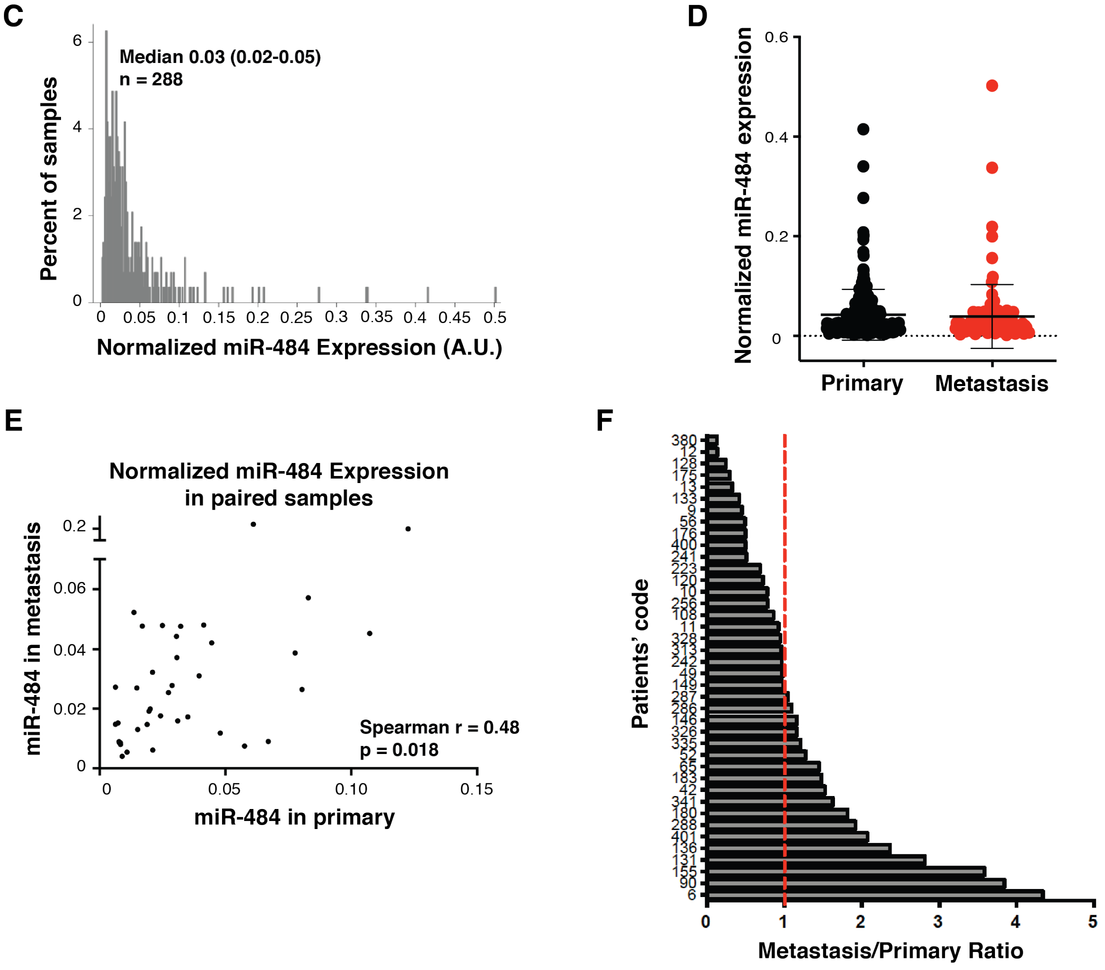

Figure S6. Analyses of microRNAs expression in the MITO16A-MANGO OV2 trial.

A. Table reporting the normalized expression (mean and standard deviation) of the indicated microRNAs (miR) in the analyzed samples. The number of positive samples over the total number of analyzed samples is reported in the last column.

B. Schematic representation of the reported role of miR-484 in the regulation of angiogenesis in ovarian cancer (see reference 4 in the main text)

C. Graph reporting distribution of analyzed samples based on the normalized expression of miR-484.

D. Dot plot reporting normalized expression of miR-484 in primary and metastatic lesions.

E. Graph reporting the correlation of miR-484 expression in paired primary and metastatic lesions.

E. Graph reporting the ratio of miR-484 expression in paired primary and metastatic lesions.

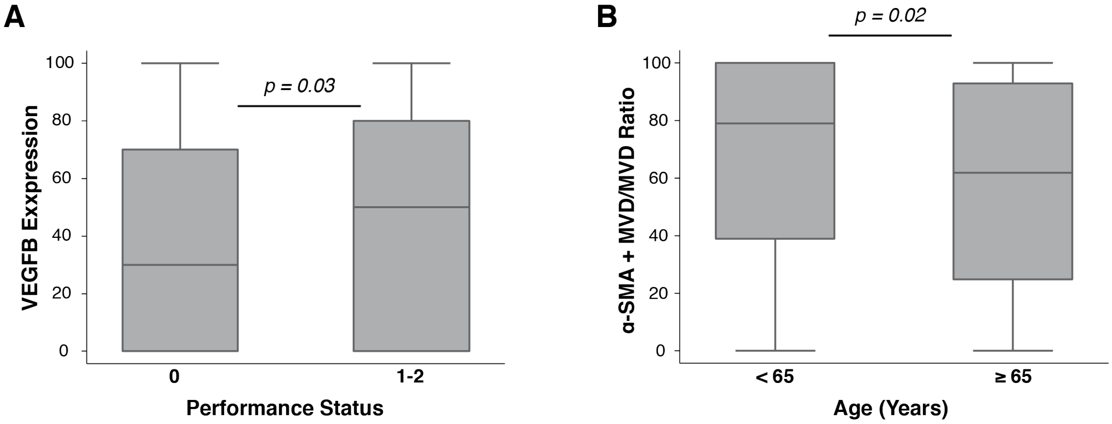

Figure S7. Analyses of biomarkers expression with clinic-pathological variables of patients enrolled in the MITO16A-MANGO OV2 trial.

A. Graph reporting the expression of VEGFB in patients divided based on their ECOG performance status, as indicated. Significance is reported in the figure.

B. Graph reporting the expression of α-SMA+MVD/MVD ratio in patients divided based on their age, as indicated. Significance is reported in the figure.

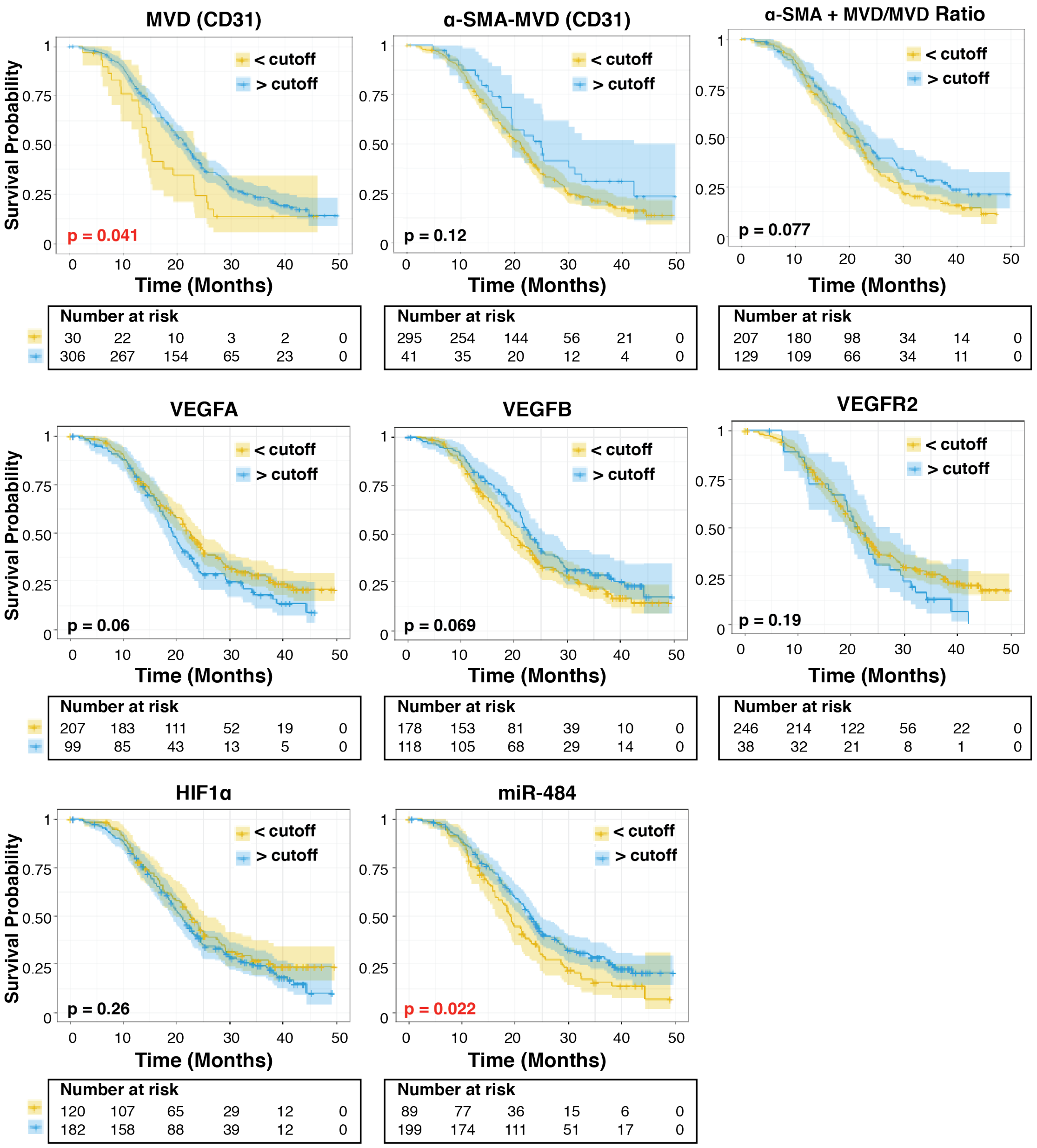

Figure S8. Kaplan Meier curves evaluating the progression free survival of patients enrolled in the MITO16A-MANGO OV2 trial.

Kaplan Meier analyses evaluating the progression free survival of MITO16A-MANGO OV2 patients divided based on the expression of the indicated biomarkers. Significance (p value) is reported in each graph. Number of patients at risk are reported under each graph.

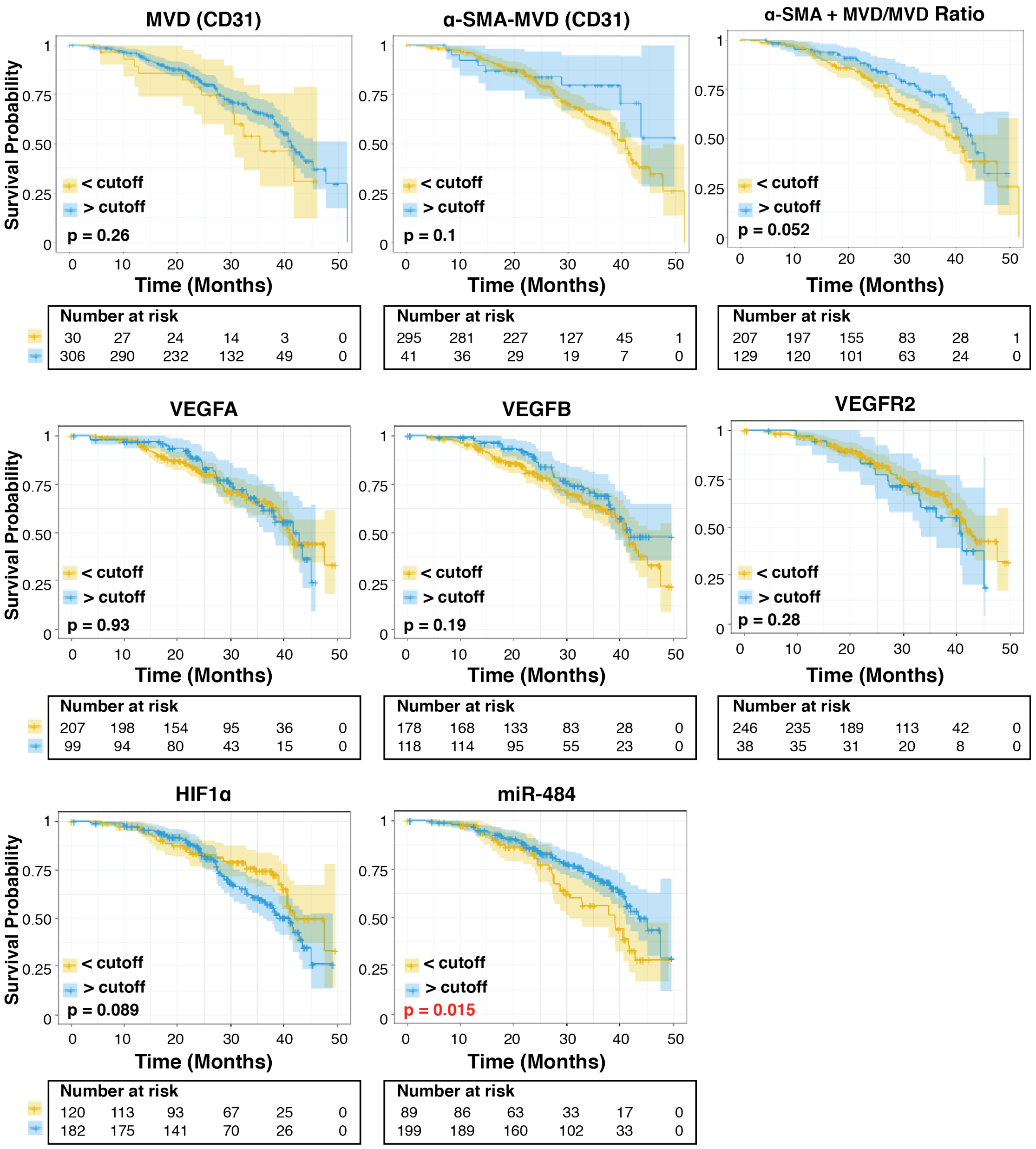

Figure S9. Kaplan Meier curves evaluating the overall survival of patients enrolled in the MITO16A-MANGO OV2 trial.

Kaplan Meier analyses evaluating the overall free survival of MITO16A-MANGO OV2 patients divided based on the expression of the indicated biomarkers. Significance (p value) is reported in each graph. Number of patients at risk are reported under each graph.

Table S1: Analysis of biomarkers (continuous and dummy for zero variables) adjusted for PFS and OS.

	PFS			OS		
	**HR**	**(95% CI)**	**P**	**HR**	**(95% CI)**	**P**
**MVD:**						
Continuous variable	1.00	(0.99-1.00)	0.328	1.00	(0.99-1.01)	0.857
**α-SMA+MVD:**						
Continuous variable	1.00	(0.99-1.00)	0.729	1.00	(0.99-1.01)	0.503
**Ratio:**						
*Continuous variable	0.97	(0.69-1.35)	0.836	0.74	(0.45-1.21)	0.232
**MIR 484:**						
Continuous variable	0.17	(0.01-2.64)	0.206	0.01	(0.001-3.37)	0.128
**VEGFA:**						
Continuous variable	1.00	(1.00-1.01)	0.284	1.00	(0.99-1.01)	0.968
**VEGFB:**						
Continuous variable	1.00	(0.99-1.01)	0.984	1.00	(0.99-1.01)	0.477
Dummy variable for zero	1.12	(0.68-1.85)	0.662	0.78	(0.39-1.57)	0.488
**VEGFR2:**						
Continuous variable	1.01	(1.00-1.02)	0.098	1.01	(1.00-1.03)	0.051
Dummy variable for zero	1.62	(0.86-3.06)	0.133	3.00	(0.98-9.20)	0.055
**HIF1a:**						
Continuous variable	1.00	(0.99-1.01)	0.498	1.00	(0.99-1.01)	0.797
Dummy variable for zero	0.75	(0.47-1.19)	0.222	0.65	(0.33-1.28)	0.215

Legend to Table S1:

HR = Hazard ratio

CI = Confidence Interval

MVD = Micro Vessel Density (CD31+)

* the model testing the effect of the α-SMA+MVD Ratio was also adjusted for MVD continuous variable

Table S2: Analysis of biomarkers best cutoff for PFS and OS, original and shrunken coefficients.

	PFS					
	Original coefficients			Shrunken coefficients		
	HR	(95% CI)	P	HR	(95% CI)	P
**MVD:**						
> 31.2	0.65	(0.43-0.99)	**0.043**	0.72	(0.38-1.39)	0.332
**α-SMA+MVD:**						
> 64.1	0.72	(0.48-1.09)	0.118	0.82	(0.36-1.91)	0.649
**Ratio:**						
> 0.86	0.79	(0.61-1.03)	0.077	0.85	(0.41-1.77)	0.667
**miR-484:**						
> 0.017	0.72	(0.54-0.95)	**0.023**	0.76	(0.40-1.47)	0.421
**VEGFA:**						
> 56.7	1.31	(0.99-1.73)	0.060	1.21	(0.76-1.93)	0.422
**VEGFB:**						
> 45.0	0.77	(0.59-1.02)	0.070	0.84	(0.42-1.67)	0.612
**VGFR2:**						
> 60.0	1.29	(0.89-1.87)	0.186	1.11	(0.67-1.84)	0.676
**HIF1a:**						
> 0.0	1.17	(0.89-1.54)	0.259	1.03	(0.61-1.74)	0.898
	OS					
	HR	(95% CI)	P	HR	(95% CI)	P
**MVD:**						
> 31.2	0.72	(0.4-1.28)	0.266	0.94	(0.16-5.66)	0.945
**α-SMA+MVD:**						
> 64.1	0.57	(0.29-1.13)	0.108	0.71	(0.12-4.11)	0.702
**Ratio:**						
> 0.86	0.68	(0.46-1.01)	0.054	0.75	(0.21-2.71)	0.667
**miR-484:**						
> 0.017	0.61	(0.40-0.91)	**0.016**	0.66	(0.19-2.32)	0.517
**VEGFA:**						
> 56.7	0.98	(0.64-1.49)	0.926	0.98	(0.42-2.31)	0.964
**VEGFB:**						
> 45.0	0.76	(0.50-1.15)	0.192	0.89	(0.2-4.09)	0.884
**VGFR2:**						
> 60.0	1.33	(0.79-2.25)	0.286	1.03	(0.18-5.91)	0.969
**HIF1a:**						
> 0.0	1.43	(0.94-2.17)	0.091	1.26	(0.28-5.63)	0.760

MITO-16 - MANGO-OV2
First line protocol

A MULTICENTER STUDY IN PATIENTS WITH STAGE III-IV EPITHELIAL OVARIAN CANCER TREATED WITH CARBOPLATIN/PACLITAXEL WITH BEVACIZUMAB: CLINICAL AND BIOLOGICAL PROGNOSTIC FACTORS

EudraCT number: 2012-003043-29

**Sponsor non-profit:**	National Cancer Institute, Naples, Italy
**Principal Investigators:**	Sandro Pignata, Nicoletta Colombo
**Study Coordinators:**	Francesco Perrone, Gennaro Daniele, Roldano Fossati
**Statistician:**	Ciro Gallo, Irene Floriani
**Steering Committee:**	Sandro Pignata, Francesco Perrone, Nicoletta Colombo, Roldano Fossati, Ciro Gallo, Irene Floriani, Domenica Lorusso, Gennaro Daniele
**Research Nurse:**	Jane Bryce
**Data Manager:**	Cristiana De Luca
**Coordinating centres**:	Clinical Trials Unit - National Cancer Institute, Naples (MITO); Clinical Trials Lab – Mario Negri Institute, Milan (MANGO)

Ethics Committee approval Version 0, date: 18/07/2012

Administrative approval: 24/09/2012

AMENDMENTS

Number	Type	Description
1	Substantial	Amendment to the translational study

Protocol Authorization

I have read this study protocol and agree that it contains all the information required to conduct the study. I agree to conduct the study as set out in this protocol. In particular, I agree to adhere to the moral, ethical and scientific principles governing clinical research as set out in the Declaration of Helsinki, the guidelines on Good Clinical Practice and the appropriate national laws.

Trial Promoter Coordinating Centre

Dr. Francesco Perrone	Date

TABLE OF CONTENTS

1. BACKGROUND 6
1.1 Current first-line treatment of ovarian cancer 6
1.2 Bevacizumab 6
1.2.1 Safety of bevacizumab 8
1.2.2 Potential Predictive Factors of efficacy of Bevacizumab 11
2. STUDY OBJECTIVES 14
3. STUDY DESIGN 15
3.1 Rationale for study design 15
3.1.1 Planned Treatment 15
3.1.2 Rationale for dose selection 15
3.2 Number of Patients 15
3.3 Centres 15
4. PATIENT SELECTION 16
5. TREATMENT PLAN 19
6. DOSING AND SCHEDULING OF STUDY DRUGS 20
6.1 Bevacizumab 20
6.1.1 Dose modifications and delays 21
6.1.2 Management of bevacizumab related to potential adverse events 23
6.1.3 Formulation, Packaging and Labelling 27
6.2 Carboplatin 27
6.2.1 Carboplatin dose 28
6.3 Paclitaxel 28
6.3.1 Paclitaxel dose 29
6.4 DOSE MODIFICATIONS AND DELAYS OF PACLITAXEL AND CARBOPLATIN 29
6.4.1 Minimum requirements for retreatment 29
6.4.2 Dose modifications and delays of carboplatin and paclitaxel 29
6.5 Concomitant Medications 30
6.6 Criteria for Premature Withdrawal 31
6.6.1 Withdrawal of patients from the biomarker programme 32
7. ASSESSMENTS AND PROCEDURES DURING THE STUDY TREATMENT 33

7.1 Screening Examination and Eligibility Screening Form 33
7.2 Procedures for Enrolment of Eligible Patients 33
7.3 Clinical Assessments and Procedures 33
7.3.1 Tumour response criteria 33
7.3.2 Measurability of tumour lesions 34
7.3.3 Identification of "Target" and "Non-Target" lesions 34
7.3.4 Evaluation of target lesion response 36
7.3.5 Evaluation of non target lesion response 36
7.3.6 Evaluation of best overall response according to RECIST 37
7.3.7 Clinical safety assessments 38
8. BIOMARKER SAMPLES AND ASSESSMENTS 43
8.1.1 Specimen Types 43
9. ADVERSE EVENTS REPORTING 44
9.1 Definitions 44
9.2 Collection and reporting of adverse events 45
9.3 Collection and reporting of serious adverse events (SAE) 45
9.4 Causality assessment between treatment and event 46
9.5 Procedures for safety reporting 48
10. STATISTICAL ANALYSIS 50
11. QUALITY ASSURANCE AND MONITORING 50
12. DATA COLLECTION PROCEDURES 50
13. ADMINISTRATIVE ASPECTS 51
14. COORDINATING CENTER CONTACTS 52
15. REFERENCES 54
16. APPENDIX 1: BEVACIZUMAB LABELING 58
17. APPENDIX 2: SAMPLES HANDLING AND SHIPPING 58
Samples for circulating plasma biomarkers (including miRNA): 59
18. APPENDIX 3: TRANSLATIONAL PROJECT 60

1. BACKGROUND

Epithelial Ovarian Cancer (EOC) is the 7th most common cause of cancer death for women worldwide. In 2012, 22.280 new diagnosis and 15.500 deaths for EOC have been estimated in US, where the EOC is the 5th most common cause of cancer-related death among women[1]. Despite improvements in the treatment of ovarian cancer, only modest increases in overall survival (OS) have been registered[2,3]. In fact, it has improved by only 5%, from 20% to only 25%, for women with advanced-stage tumours and as such, mortality remains high. This is partly due to the fact that ovarian cancer is frequently not diagnosed until it has progressed to an advanced stage. Ovarian cancer is considered a chemo-responsive neoplasm, with initial response rates to systemic chemotherapy exceeding 80% when integrated with primary cytoreductive surgery[4]. Despite this, the majority of patients who achieve a complete response (CR) with front-line chemotherapy ultimately develop recurrent disease, with over 50% of women diagnosed with epithelial ovarian cancer eventually dying from their disease[5]. Major trials published over the past 15 years report that the median progression free survival (PFS) for patients with advanced disease ranges between 16 and 23 months while the median OS lies between 31 and 65 months[6-10].

Since the introduction of platinum-based chemotherapy, further advances in treatment have been modest. Survival rates of patients with advanced, recurrent or relapsed ovarian cancer remain poor and there continues to be a significant unmet medical need for improved treatment regimens. In this regard, molecular targeted therapeutic agents herald a new era for cancer treatment. In the setting of epithelial ovarian cancer, a growing body of evidence supports the use of anti-angiogenic agents in combination with chemotherapies[11]. In particular, bevacizumab (Avastin®), a monoclonal antibody targeted against the pro-angiogenic vascular endothelial growth factor (VEGF), holds significant therapeutic potential.

1.1 Current first-line treatment of ovarian cancer

Debulking surgery consisting in laparotomy with total abdominal hysterectomy, bilateral salpingo-oophorectomy and omentectomy is considered the first therapeutic step for the ovarian cancer patients. Systemic chemotherapy with carboplatin and paclitaxel represents the standard first line treatment for high risk early-stage (stage IA-B grade 3, IC stage or clear cell histology any grade) and advanced stage ovarian cancer patients [12].

One of the most disappointing issues of the ovarian cancer treatment is that, even in case of response to first-line platinum-based therapy, the chance to experience a recurrence/progression is very high and the probability to respond to further treatments decrease proportionally. Major efforts have been made recently to try to foster new treatments for ovarian cancer patients.

1.2 Bevacizumab

Bevacizumab is a recombinant humanized monoclonal antibody to Vascular Endothelial Growth Factor (VEGF) composed of human IgG1 framework regions and antigen-binding complementary determining regions from a murine monoclonal antibody (muMAb VEGF A.4.6.1) that blocks the binding of human VEGF to all VEGF-A receptors[13].

Bevacizumab recognizes and neutralizes isoforms of VEGF with a K_d of around 8×10^{-10} M. It does not recognize other peptide growth factors tested (fibroblast growth factor, epidermal growth factor, hepatocyte growth factor, platelet-derived growth factor and nerve growth factor). It may exert a direct anti-angiogenic effect by binding to and clearing VEGF from the tumour environment. Additional anti-tumour activity may be obtained via the effects of bevacizumab on tumour vasculature, interstitial pressure and blood vessel permeability, providing for enhanced chemotherapy delivery to tumour cells[14]. In addition, bevacizumab showed synergistic antiangiogenic activity with docetaxel, as assessed by endothelial cell proliferation and tubule formation, *in vitro*[15].

Anti-VEGF antibodies have shown benefit when combined with chemotherapy in preclinical models of different tumour types. Bevacizumab can block the growth of a number of human cancer cell lines grown in nude mice, including metastatic colorectal cancer (mCRC), non-squamous non-small cell lung cancer (NSCLC), metastatic or locally recurrent breast cancer (BC), prostate cancer, head and neck cancer and metastatic renal cell carcinoma (mRCC) [16-18].

Based on the results of two randomised trials, bevacizumab has been cleared in combination with chemotherapy as first line treatment for advanced ovarian cancer patients.

The first trial, GOG218, was an international, multicenter, randomized, placebo-controlled trial of paclitaxel and carboplatin with or without bevacizumab (15 mg/kg i.v.) in first-line treatment of 1873 patients with stage III/IV epithelial ovarian cancer, primary peritoneal cancer or fallopian tube cancer [19]. Patients who had completed primary maximal debulking surgery in the previous 12 weeks were randomised to one of three different treatment arms. Patients in arm 1 (control) received carboplatin plus paclitaxel for 6 cycles, those in arm 2 received the same regimen for 6 cycles plus bevacizumab (15mg/kg q3w) for 5 cycles (from cycle 2 to 6), whereas patients in arm 3 received the same chemotherapy combination for 6 cycles and bevacizumab (15mg/kg q3w) for 22 cycles. The two bevacizumab containing arms have been both compared with control. The PFS was the primary end-point. A significant PFS improvement was observed for arm 3 compared with control (hazard ratio 0.717; 95%CI 0.625 to 0.824; $P<0.0001$). In contrast, there was no significant improvement of PFS in arm 2 vs arm 1 [19].

The second trial, ICON7, was a randomized Phase III trial that recruited 1528 patients with high-risk early stage disease or advanced stage ovarian carcinoma. Patients received conventional carboplatin plus paclitaxel for 6 cycles, or the same treatment plus bevacizumab (7.5 mg/kg q3w) during chemotherapy, with bevacizumab monotherapy continuing for an additional 36 weeks. In this open-label study, the results were broadly supportive of the results from GOG218 with an improvement of PFS (hazard ratio 0.87; 95%CI 0.77 to 0.99; $P = 0.04$) [20].

Interestingly, according the data, the ovarian cancer patients who might mostly benefit from the bevacizumab addition are those with poor prognosis (stage III and IV FIGO represents the overall population in the GOG218 and the prevalent one in the ICON7 with the high risk early stage patients).

1.2.1 Safety of bevacizumab

Exhaustive safety information on bevacizumab can be found in Summary of Product Characteristics. In the Phase III study programme conducted across several tumours, bevacizumab has demonstrated an acceptable safety profile with a relatively low incidence of grade 3 or 4 adverse events (AEs). Specific side effects associated with the use of bevacizumab (either alone or in combination with chemotherapy) are as follows:

Gastrointestinal perforation: Bevacizumab has been associated with serious cases of gastrointestinal perforation or fistulae in 2.4% of patients (versus 0.3% in controls), with about one-third of cases being fatal (0.2%-1% of all bevacizumab treated patients). The typical presentation may include abdominal pain, nausea, emesis, constipation, and fever. Perforation can be complicated by intra-abdominal abscess and fistula formation. The majority of cases occurred within the first 50 days of therapy. In some cases underlying intra-abdominal inflammation was present (either from gastric ulcer disease, tumour necrosis, diverticulitis or chemotherapy-associated colitis) caution should be exercised when treating such patients with bevacizumab. In phase II trials in ovarian cancer, gastrointestinal perforation was most associated with refractory or resistant ovarian cancer and greater cumulative use of chemotherapy[37]. In contrast, no case of gastrointestinal perforation has been reported in either of the Phase II trials which evaluated bevacizumab in the front-line setting[21,22]. In the recently completed phase III GOG218 trial[19], gastrointestinal perforation, fistula, necrosis or leak occurred in 2.6% of those patients receiving 22 cycles of bevacizumab compared with 1.2% of those who received paclitaxel and carboplatin without bevacizumab. In the ICON 7, the rate of GI perforation G≥3 was significantly worse in the bevacizumab-containing arm (1.4% vs 0.4%) compared with the non bevacizumab-containing arm[20]. Bevacizumab should be permanently discontinued in patients who develop gastrointestinal perforation.

Fistulae:Patients may be at increased risk for the development of fistulae when treated with Bevacizumab. Permanently discontinue Bevacizumab in patients with TE (tracheoesophageal) fistula or any grade 4 fistula. Limited information is available on the continued use of Bevacizumab in patients with other fistulae. In cases of internal fistula not arising in the GI tract, discontinuation of Bevacizumab should be considered

Wound-healing complications: Bevacizumab may adversely affect the wound healing process. Therapy should not be initiated for at least 28 days following major surgery or until the surgical wound is fully healed. In patients who experienced wound healing complications during therapy, treatment should be withheld until the wound is fully healed. Therapy should be withheld for elective surgery.

Hypertension: An increased incidence of hypertension was observed in Bevacizumab-treated patients. Clinical safety data suggest that the incidence of hypertension is likely to be dose-dependent. Pre existing hypertension should be adequately controlled before starting Bevacizumab treatment. There is no information on the effect of Bevacizumab in patients with uncontrolled hypertension at the time of initiating therapy. Monitoring of blood

pressure is generally recommended during therapy. In most cases hypertension was controlled adequately using standard antihypertensive treatment appropriate for the individual situation of the affected patient. The use of diuretics to manage hypertension is not advised in patients who receive a cisplatin-based chemotherapy regimen. Bevacizumab should be permanently discontinued if medically significant hypertension cannot be adequately controlled with antihypertensive therapy, or if the patient develops hypertensive crisis or hypertensive encephalopathy.

Reversible posterior leukoencephalopathy syndrome (RPLS): There have been rare reports of Bevacizumab-treated patients developing signs and symptoms that are consistent with Reversible Posterior Leukoencephalopathy Syndrome (RPLS), a rare neurologic disorder, which can present with the following signs and symptoms among others: seizures, headache, altered mental status, visual disturbance, or cortical blindness, with or without associated hypertension. A diagnosis of RPLS requires confirmation by brain imaging. In patients developing RPLS, treatment of specific symptoms including control of hypertension is recommended along with discontinuation of Bevacizumab. The safety of reinitiating Bevacizumab therapy in patients previously experiencing RPLS is not known.

Proteinuria: Patients with a history of hypertension may be at increased risk for the development of proteinuria when treated with Bevacizumab. There is evidence suggesting that all grade proteinuria may be related to the dose. Monitoring of proteinuria by dipstick urinalysis is recommended prior to starting and during therapy. Therapy should be permanently discontinued in patients who develop Grade 4 proteinuria (nephrotic syndrome).

Thromboembolism: In clinical trials, the incidence of arterial thromboembolic events including cerebrovascular accidents (CVAs), transient ischaemic attacks (TIAs) and myocardial infarctions (MIs) was higher in patients receiving Bevacizumab in combination with chemotherapy compared to those who received chemotherapy alone. Patients receiving Bevacizumab plus chemotherapy, with a history of arterial thromboembolism or age greater than 65 years have an increased risk of developing arterial thromboembolic events during therapy. Caution should be taken when treating these patients with Bevacizumab. herapy should be permanently discontinued in patients who develop arterial thromboembolic events. Patients may be at risk of developing venous thromboembolic events, including pulmonary embolism under Bevacizumab treatment. Bevacizumab should be discontinued in patients with life-threatening (Grade 4) thromboembolic events, including pulmonary embolism. Patients with thromboembolic events ≤Grade 3 need to be closely monitored.

Haemorrhage: Patients treated with Bevacizumab have an increased risk of haemorrhage, especially tumour-associated haemorrhage. Bevacizumab should be discontinued permanently in patients who experience Grade 3 or 4 bleeding during Bevacizumab therapy (see section 4.8). Patients with untreated CNS metastases were routinely excluded from clinical trials with Bevacizumab, based on imaging procedures or signs and symptoms. Therefore, the risk of CNS haemorrhage in such patients has not been prospectively evaluated in randomised clinical trials. Patients should be monitored for signs and symptoms of CNS bleeding, and Bevacizumab treatment discontinued in cases of intracranial bleeding. There is no information on the safety profile of Bevacizumab in patients with congenital bleeding diathesis, acquired coagulopathy or in patients receiving

full dose of anticoagulants for the treatment of thromboembolism prior to starting Bevacizumab treatment, as such patients were excluded from clinical trials.Therefore, caution should be exercised before initiating therapy in these patients. However, patients who developed venous thrombosis while receiving therapy did not appear to have an increased rate of grade 3 or above bleeding when treated with a full dose of warfarin and Bevacizumab concomitantly. Patients with non-small cell lung cancer treated with Bevacizumab may be at risk of serious, and in some cases fatal, pulmonary haemorrhage/haemoptysis. Patients with recent pulmonary haemorrhage/ haemoptysis (> 2.5 ml of red blood) should not be treated with Bevacizumab.

Congestive heart failure: declines in left ventricular ejection fraction to symptomatic CHF, requiring treatment or hospitalisation. Caution should be exercised when treating patients with clinically significant cardiovascular disease such as pre-existing coronary artery disease, or congestive heart failure with Bevacizumab. Most of the patients who experienced CHF had metastatic breast cancer and had received previous treatment with anthracyclines, prior radiotherapy to the left chest wall or other risk factors for CHF were present.In patients in AVF3694g who received treatment with anthracyclines and who had not received anthracyclines before, no increased incidence of all grade CHF was observed in the anthracycline + bevacizumab group compared to the treatment with anthracyclines only. CHF grade 3 or higher events were somewhat more frequent among patients receiving bevacizumab in combination with chemotherapy than in patients receiving chemotherapy alone. This is consistent with results in patients in other studies of metastatic breast cancer who did not receive concurrent anthracycline treatment.

Neutropenia and infections: Increased rates of severe neutropenia, febrile neutropenia, or infection with or without severe neutropenia (including some fatalities) have been observed in patients treated with some myelotoxic chemotherapy regimens plus Bevacizumab in comparison to chemotherapy alone. This has mainly been seen in combination with platinum- or taxane-based therapies in the treatment of NSCLC and mBC while was not observed in the GOG 218 and ICON7 trials in ovarian cancer.

Hypersensitivity reactions/infusion reactions: Patients may be at risk of developing infusion/hypersensitivity reaction. Close observation of the patient during and following the administration of bevacizumab is recommended as expected for any infusion of a therapeutic humanized monoclonal antibody. If a reaction occurs, the infusion should be discontinued and appropriate medical therapies should be administered. A systematic premedication is not warranted.

Osteonecrosis of the jaw: Cases of ONJ have been reported in cancer patients treated with Bevacizumab, the majority of whom had received prior or concomitant treatment with i.v. bisphosphonates, for which ONJ is an identified risk. Caution should be exercised when Bevacizumab and i.v. bisphosphonates are administered simultaneously or sequentially. Invasive dental procedures are also an identified risk factor. A dental examination and appropriate preventive dentistry should be considered prior to starting the treatment with Bevacizumab. In patients who have previously received or are receiving i.v. bisphosphonates invasive dental procedures should be avoided, if possible.

Eye disorders*:* Adverse reactions have been reported from unapproved intravitreal use. These reactions included infectious endophthalmitis, intraocular inflammation such as sterile endophthalmitis, uveitis and vitritis, retinal detachment, retinal pigment epithelial tear, intraocular pressure increased, intraocular haemorrhage such as vitreous

haemorrhage or retinal haemorrhage and conjunctival haemorrhage. Some of these appeared as serious adverse reactions.

Ovarian failure/fertility: Bevacizumab may impair female fertility. Therefore fertility preservation strategies should be discussed with women of child-bearing potential prior to starting treatment with Bevacizumab.

1.2.2 Potential Predictive Factors of efficacy of Bevacizumab

1.2.2.1 Hypertension

Arterial hypertension has been invariably reported as one of the most frequent side effects of bevacizumab administration. The reported frequency of bevacizumab-induced hypertension lays around 18% - 34% (*Summary of Product Characteristics*), seems to be dose-dependent and largely rely on the definition of hypertension and the reporting of this. Indeed, since in most of the trials the G2 hypertension is not mandatory to be reported, the actual rate of hypertension during the administration of bevacizumab might be higher.

The mechanism by which antiangiogenic drugs lead to hypertension is unknown, albeit several hypotheses have been proposed during the recent years. The mechanisms proposed encompass the decreased VEGF-induced (NO) synthesis and the increased arterial resistance due to the decreased number of capillary beds as well as the endothelial dysfunction due to direct effect of bevacizumab onto endothelial cells [23,24] .

Interestingly, several early clinical data from single arm studies suggested a potential role of bevacizumab-induced hypertension as predictive factor of clinical benefit [25,26]. In fact, either the development of new hypertension or the increase in antihypertensive medications were associated with extended PFS and OS.

Subsequently, these observations were tested in retrospective analyses of phase III randomized trials. In first-line breast metastatic cancer patients treated with bevacizumab plus paclitaxel the presence of G3-G4 hypertension was found associated with increased duration of OS as compared with that in patients do not experiencing hypertension. [27]

In the phase III trial exploring the combination of bevacizumab and carboplatin-paclitaxel the median OS was significantly longer (15.9 vs 11.5 months) for those treated with bevacizumab and experiencing hypertension compared with whose not [28].

Finally, hypertension G2-3 was associated with longer PFS and OS ($p<0.001$) in RCC patients receiving bevacizumab treatment in first-line compared with those without hypertension [29].

In contrast, a meta-analysis, presented at ASCO 2010 meeting, gathering data from six trials with colorectal cancer patients treated with bevacizumab found a positive correlation between hypertension and clinical benefit only in one study [30].

1.2.2.2 Circulating Factors

The characterisation of bevacizumab mechanism of action in preclinical models, albeit largely partial, fostered in the recent years the exploration of several circulating factors as potential predictive of the bevacizumab benefit and ideally to select the patients who might benefit from a treatment.

Unfortunately, evidence gathered until now is not strong enough to prompt the the use in clinical practice of them.

Intuitively, in the field of antiangiogenic treatment the first and, up to now, the most extensively explored biomarker is VEGF[31]. Circulating baseline levels of VEGF are elevated in several tumours, especially in advanced stage, and are reported to correlate with prognosis of patients[32]. Early exploration of plasma VEGF-A as a biomarker failed to reveal any predictive role. VEGF-A appeared to have a prognostic role, with high VEGF-A levels consistently associated with poorer prognosis across tumor types explored[33]. With the recent development of a novel ELISA assay, which shows a higher preference for shorter VEGF-A isoforms, the question of a predictive effect of VEGF-A has received renewed interest. However, a recent report on this method suggested that preferential detection of the short isoform of VEGFA was not required to assess outcome correlations [34]. Plasma VEGF-A levels appeared to correlate with bevacizumab effect size in metastatic breast[35], pancreatic [36], and gastric cancers [37,38]. High VEGF-A was associated with greater PFS benefit, and in some cases OS benefit, in analyses of plasma samples from randomized trials in these tumor types[39] .Recently, changes in levels of VEGF have been reported after the treatment with bevacizumab and other antiangiogenic drugs [40-42], with the direction of the changes varying according the procedure used to measure the levels [43].

Recently published prospective exploratory biomarker analysis in a phase III study evaluating addition of bevacizumab to chemotherapy in gastric cancer give some hints of the prognostic role of VEGF-A plasma and NRP-1 concentration at baseline, albeit no convincing evidence supporting clinical routine implementation of these assays [38].

More recently the exploratory biomarker analyses of a randomised phase 3 study in HER2 negative metastatic breast cancer patients treated with or without bevacizumab in combination with paclitaxel based chemotherapy [44]. These results highlighted the weak potential of pVEGFA as predictive biomarker and suggested a potential role for pVEGFR-2.Similar changes have been described for other circulating proteins putatively related to the VEGF pathway including, TGF-alpha, E-selectin, bFGF, ICAM-1 sVEGFR1 and 2, V-CAM-1, IL6, IL8, SDF-1alpha, MIG, MIP1 alpha and beta, GM-CSF, G-CSF an others [45]. Among these, only for ICAM-1(Intra-Cellular Adhesion Molecule-1) a potential role predictive of survival benefit has been described [46] in NSCLC patients treated in a phase 3 trial.

1.2.2.3 Circulating Endothelial Cells

Recruiting endothelial cells is one of the mechanism upon which rely the entire process of angiogenesis[47]. Therefore the count of circulating endothelial cells (CEC) or progenitors

(CEP) is thought to be predictive of the antiangiogenics efficacy. Several experimental evidence supported in the last recent years this theory [48], however other reports assigned to CEC and CEP mostly a prognostic value [49].

1.2.2.4 *In-situ biomarkers*

Major difficulties have been encountered so far in selecting and evaluating potential *in-situ* biomarkers of efficacy for the antiangiogenic therapy.

First, very often the availability of the samples is very limited and in the vast majority of the cases they only come from the primary tumour and not the metastases. Second, the angiogenesis is a very complex process and underlines most advanced tumours, thus the clinical relevant differences in expression of particular factors are likely to be missed by the current methods.

However, many factors have been investigated for their potential predictive role in several clinical trials. Among these VEGFR1 and 2, P-AKT, P-MAPK, HER2, P53, Micro-vessel Density (MVD), CD31. Unfortunately, a positive, significant association with a clinical endpoint has not been described for anyone of these [45].

1.2.2.5 *Pharmacogenetic biomarkers*

Evidence gathered in various tumours, such as breast [50]**,** ovarian [51], NSCLC [52] and pancreas [53], suggested, though exploratory, a potential role of some Single Nucleotide Polymorphisms (SNPs) in predicting clinical outcome of bevacizumab based therapy as well as hypertension development during the treatment. In particular a potential association of two polymorphisms in VEGF gene was found with OS in E2100 trial [50] whilst two other polymorphisms of the same gene were found to correlate with lower rate of hypertension. These polymorphisms have not been confirmed in NSCLC patients[52]. In ovarian cancer patients, several SNPs in the Il-8, CXCR2 and VEGF genes, have been reported, in a phase II trial, to correlate with a better response rate and PFS [51]. Finally, SNPs in VEGFR-1, were found potentially predictive of a better OS and PFS [53] in favour of those patients receiving bevacizumab compared with control arm. As we noted above all these analyses are exploratory and do not allow any conclusion regarding the clinical impact of these polymorphisms. However they represents the theoretical basis to prospectively explore genetic variants in both VEGF and related pathways' genes in order to select patients who might benefit or at risk to develop toxicity with bevacizumab.

2. STUDY OBJECTIVES

Primary objective

- To explore whether clinical and biological factors, as detailed below, are able to identify a subset of patients with better prognosis in term of progression free survival (PFS) and overall survival (OS) after combined therapy with chemotherapy and bevacizumab.

Secondary objectives

- To describe the safety of bevacizumab added to carboplatin-paclitaxel chemotherapy in first line treatment of epithelial ovarian, fallopian tube and primary peritoneal cancer patients in the clinical routine practice

- To investigate the prognostic value for hypertension, circulating and in-situ biomarkers for advanced epithelial ovarian, fallopian tube and primary peritoneal cancer patients treated with carboplatin-paclitaxel and bevacizumab

- To describe the prevalence of use of oral antidiabetic therapy and antithrombotic therapy among the patients enrolled in the trial.

3. STUDY DESIGN

This is a single-arm, open-label, non-comparative, multicenter, phase IV study.

3.1 Rationale for study design

Based on the results of two randomized phase III trials [19,20], Bevacizumab has been recently approved by regulatory agencies in US and Europe as front-line treatment, in combination with carboplatin and paclitaxel, for the front-line treatment of advanced (FIGO stages III B, III C and IV) epithelial ovarian, fallopian tube, or primary peritoneal cancer. However, the magnitude of the benefit observed was not equal among all the patients in study. To now no robust data exist regarding neither prognostic nor predictive factors for ovarian cancer patients treated with bevacizumab.

The aim of this study is to collect data regarding potential clinical and molecular prognostic factors, safety and activity of bevacizumab in a population of advanced ovarian cancer patients treated with bevacizumab within the general practice.

3.1.1 Planned Treatment

Patients will receive a combination of bevacizumab, paclitaxel and carboplatin as first line treatment (for details see section 5)

3.1.2 Rationale for dose selection

Since this is an *in-label* trial, dose and schedule considered will be the approved ones as indicated by the label.

3.2 Number of Patients

This is an exploratory study and no a priori hypothesis is defined to calculate the sample size of the trial. With a sample size of 400 patients and after the registration of 280 events (either for PFS or OS), the study will have 80% power to identify a prognostic factor able to select a favourable subgroup with a 0.60 HR, for a presumed expression of the favourable prognostic factors in 20% of the population, with alpha level of 0.01[54].

Therefore, we plan to recruit approximately 400 patients over a planned recruitment period of 24 months.

3.3 Centres

This is a multicentre national study to be conducted in approximately 60 centres coordinated by MITO and MANGO groups in Italy.

4. PATIENT SELECTION

Inclusion Criteria

- Female patients ≥18 years of age.
- Patients with histologically confirmed epithelial ovarian carcinoma, fallopian tube carcinoma or primary peritoneal carcinoma, including mixed Mullerian Tumours

  Or

  Recurrent early stage epithelial ovarian or fallopian tube carcinoma treated with surgery alone.
- FIGO stage IIIB & C or IV
- ECOG Performance Status of 0–2.
- Life expectancy of at least 12 weeks.
- Signed informed consent obtained prior to initiation of any study-specific procedures and treatment as confirmation of the patient’s awareness and willingness to comply with the study requirements.
- Availability of tumour samples for molecular analyses

Exclusion Criteria

Cancer related

- Ovarian tumours with low malignant potential (i.e. borderline tumours)
- Previous systemic anti-cancer therapy for advanced ovarian cancer.
- History or evidence of brain metastases or spinal cord compression.
- History or evidence of synchronous primary endometrial carcinoma, unless all of the following criteria related to the endometrial carcinoma are met:
  - stage ≤Ia
  - no more than superficial myometrial invasion
  - no lymphovascular invasion

  - not poorly differentiated (grade 3 or papillary serous or clear cell carcinoma).

- Other malignancy within the last 5 years, except for adequately treated carcinoma in situ of the cervix or squamous carcinoma of the skin, or adequately controlled limited basal cell skin cancer.

Other-treatment related

- Any prior radiotherapy to the pelvis or abdomen.

- Surgery (including open biopsy) within 4 weeks prior to the first bevacizumab dose or planned *(In this case the patient can be enrolled but the administration of bevacizumab should be omitted at first cycle)*.

- Current or recent (within 10 days prior to the first study drug dose) use of full-dose oral or parenteral anticoagulant or thrombolytic agent for therapeutic purposes (except for central venous access patency, in which case international normalized ratio [INR] must be maintained below 1.5). Post operative prophylaxis with low molecular weight heparin sc is allowed.

- Current or recent (within 30 days of first study dosing) treatment with another investigational drug.

Laboratory related

- Inadequate bone marrow function: ANC: <1.5 x 10^9/l, or platelet count <100 x 10^9/l or Haemoglobin <9 g/dl. Patients may be transfused to maintain haemoglobin values ≥9 g/dl.

- Inadequate coagulation parameters:

  - activated partial thromboplastin time (APTT) >1.5 xULN or

  - INR >1.5

- Inadequate liver function, defined as:

  - serum (total) bilirubin >1.5 x the upper limit of normal (ULN) for the institution

  - AST/SGOT or ALT/SGPT >2.5 x ULN.

- Inadequate renal function, defined as serum creatinine >2.0 mg/dl or >177 μmol/l

- Proteinuria >1g in a 24-hour urine collection (to be performed only among patients who showed a ≥3+ at urine dipstick).

Patient related

- Pregnant or lactating patients.

- History or evidence of thrombotic or hemorrhagic disorders; including cerebrovascular accident (CVA) / stroke or transient ischemic attack (TIA) or sub-arachnoid haemorrhage within ≤6 months prior to the first study treatment).

- Uncontrolled hypertension (sustained systolic >150 mm Hg and/or diastolic >100 mm Hg despite antihypertensive therapy) or clinically significant (i.e. active) cardiovascular disease, including:

    - myocardial infarction or unstable angina within ≤6 months prior to the first study treatment

    - New York Heart Association (NYHA) grade II or greater congestive heart failure (CHF)

    - serious cardiac arrhythmia requiring medication (with the exception of atrial fibrillation or paroxysmal supraventricular tachycardia)

    - peripheral vascular disease ≥grade 3 (i.e. symptomatic and interfering with activities of daily living requiring repair or revision).

- History of abdominal fistula, gastrointestinal perforation, or intra-abdominal abscess within 6 months prior to the first study treatment.

- Non-healing wound, ulcer or bone fracture. Patients with granulating incisions healing by secondary intention with no evidence of fascial dehiscence or infection are eligible but require three weekly wound examinations.

- Evidence of any other medical conditions (such as psychiatric illness, peptic ulcer, etc.), physical examination or laboratory findings that may interfere with the planned treatment, affect patient compliance or place the patient at high risk from treatment-related complications.

5. TREATMENT PLAN

Patients will receive (in the following order):

- Bevacizumab 15 mg/kg i.v. on Day 1 every 3 weeks (+/- 3 days) until
    - disease progression

      or
    - the occurrence of an unacceptable toxicity

      or
    - until Cycle 22nd of bevacizumab.

  Bevacizumab must not be administered prior to surgery.

  Bevacizumab will start with Cycle, 1 however, if this begins less than 28 days after major surgery, in which case bevacizumab will be omitted from the first treatment cycle.

and

- Paclitaxel 175 mg/m^2 on Day 1 every 3 weeks (+/- 3 days) until
    - disease progression

      or
    - the occurrence of an unacceptable toxicity

      or
    - until completion of up to 6 cycles of chemotherapy

and

- Carboplatin (AUC 5) on Day 1 every 3 weeks (+/- 3 days) from Cycle 1 until
  - disease progression

    or
  - the occurrence of an unacceptable toxicity

    or
  - until completion of up to 6 cycles of chemotherapy.

If chemotherapy is discontinued permanently, bevacizumab may be continued, provided there is no evidence of disease progression or unacceptable toxicity.

Patient safety and adverse events will be assessed at each visit until 30 days following the last dose of bevacizumab, then every 6 months until second-line treatment start or the end of the study.

Disease progression will be assessed at the end of Cycles 3 and 6, then every 3 cycles while the patient is receiving bevacizumab maintenance and and then at cessation of bevacizumab . After cessation of bevacizumab, follow up will be performed every 3 months (see Table 5 below) until disease progression.

6. DOSING AND SCHEDULING OF STUDY DRUGS

6.1 Bevacizumab

The date of the first administration of bevacizumab must be after at least 4 weeks from last surgery. If a patient has had two operations, for example an initial operation to remove what was thought to be a benign cyst and then a second operation to formally stage and maximally debulk the ovarian tumour, then the second operation date should be documented as the date of last surgery. The date of diagnosis, however, should be recorded as the date of the initial operation where ovarian cancer was diagnosed.

The dose of 15 mg/kg of bevacizumab will be administered intravenously every 3 weeks. Bevacizumab must be administered on the same day but before paclitaxel followed by carboplatin.

The first dose of bevacizumab will be administered over 90 minutes. If the first infusion is well tolerated without infusion-related reaction (e.g. fever and/or chills), the 2nd dose will be administered over 60 minutes. If the 2nd dose is also well tolerated without an infusion reaction, all subsequent doses will be administered over 30 minutes.

6.1.1 Dose modifications and delays

No dose reduction of bevacizumab is foreseen for an individual patient. Dose omission or termination of treatment will be based on the observed toxicities. If any weight change of more than 10% is observed, the treatment dosage should be modified accordingly. Other than in cases of significant weight change, no other dose modifications are allowed for bevacizumab.

As described below, bevacizumab treatment may be either temporarily or permanently suspended in the case of hypertension, proteinuria, thrombosis/embolism, haemorrhage, CHF or wound healing complications in addition to any other serious bevacizumab-related toxicity (grade 3 or 4).

Bevacizumab should be temporarily withheld in the event of febrile grade 4 neutropenia and/or grade 4 thrombocytopenia (regardless of the relationship to treatment), since these conditions are predisposing factors for an increased bleeding tendency. In general, appropriate management for grade 3 or 4 bevacizumab-related events is described in table 1 below.

Bevacizumab omitted in Cycle 1 because of surgery within the previous 28 days will not be replaced. Bevacizumab will be withheld in case of interval debulking surgery or any other surgery to be performed since, at least, 28 days prior and up to , at least, 28 days after the surgical intervention.

The dose(s) omitted due to interval debulking or other surgery or other reasons can be replaced (i.e. not omitted), which will extend the time required to complete the maximum number of cycles foreseen.

For any further information on safety of bevacizumab are available in the Summary of Product Characteristics.

Table 1 - Management of grade 3 or 4 bevacizumab-related adverse events

**First occurrence**	Hold bevacizumab until toxicity has improved to ≤ grade 1
**Second occurrence**	Permanently discontinue bevacizumab treatment

In addition, bevacizumab treatment should be permanently discontinued in patients experiencing any of the following events:

- Reversible Posterior Leucoencephalopathy Syndrome (RPLS)
- Grade 3/4 hemorrhagic/bleeding events
- Any grade CNS bleeding
- Any grade of arterial thromboembolism (note pulmonary emboli are considered venous thromboemboli)
- Grade 4 venous thromboembolism
- Grade 4 hypertension (hypertensive crisis)
- Nephrotic syndrome
- Grade 3/4 left ventricular dysfunction (CHF)
- Any grade of gastrointestinal perforation
- Any grade of tracheo-esophageal fistula
- Grade 4 non-gastrointestinal fistula
- Any grade of hypersensitivity/allergic reactions related to bevacizumab.

6.1.2 Management of bevacizumab related to potential adverse events

6.1.2.1 CNS bleeding

Patients should be monitored for signs and symptoms of CNS bleeding, and bevacizumab treatment discontinued in case of intracranial bleeding of any grade. Patients with untreated CNS metastases were routinely excluded from clinical trials with bevacizumab, based on imaging procedures or signs and symptoms. Therefore, the risk of CNS hemorrhage in such patients has not been prospectively evaluated.

6.1.2.2 Hypertension

Patients must be closely monitored on study for the development or worsening of hypertension. Blood pressure measurements should occur after the patient has been in a resting position for ≥5 minutes. If the initial BP reading is ≥140 mmHg systolic and/or ≥90 mmHg diastolic pressures, the result should be verified with a repeat measurement. If hypertension occurs, bevacizumab treatment should be managed as described in Table 2.

Table 2 Bevacizumab treatment management for hypertension

NCI CTCAE v4.03 grade	Hypertension Pattern	Treatment Action
Grade 1	Prehypertension (systolic BP120 - 139 mm Hg or diastolic BP 80 - 89 mm Hg)	Give bevacizumab
Grade 2	Stage 1 hypertension (systolic BP 140 - 159 mm Hg or diastolic BP 90 - 99 mm Hg); medical intervention indicated;<br><br>recurrent or persistent (≥24 hrs); symptomatic increase by >20 mm Hg (diastolic) or to >140/90 mm Hg if previously within normal limits; monotherapy indicated	Withhold bevacizumab if BP >150/100 mmHg and start antihypertensive therapy. Once BP is <150/100 mmHg, patients may continue bevacizumab therapy
Grade 3	Stage 2 hypertension (systolic BP >=160 mm Hg or diastolic BP >=100 mm Hg); medical intervention indicated; more than one drug or more intensive therapy than previously used indicated	Hold bevacizumab for persistent or symptomatic hypertension and discontinue permanently if hypertension is not controlled according to Investigator judgment.
Grade 4	Life- threatening consequences (e.g. malignant hypertension, transient or permanent neurologic deficit, hypertensive crisis); urgent intervention<br><br>Indicated	Permanently discontinue bevacizumab

6.1.2.3 Proteinuria

Proteinuria will be assessed within 72 hours before each bevacizumab treatment by dipstick method unless assessed by 24-hour urine collection. Alternatively, proteinuria

testing can be performed according to local standards. An algorithm for the appropriate management following a positive dipstick result with corresponding bevacizumab treatment management guidance is provided below (Table 3):

Nephrotic syndrome: Bevacizumab must be permanently discontinued if nephrotic syndrome is detected at any time.

Table 3 Bevacizumab treatment management for proteinuria

**NCI CTCAE v4.03 grade**	**Urinalysis**	**Treatment action**
Grade 1	1+ proteinuria<br><br>urinary protein <1.0 g/24 hrs	No bevacizumab modifications
Grade 2	2+ proteinuria<br><br>urinary protein 1.0 - 3.4 g/24 hrs	Suspend bevacizumab for urine protein level ≥ 2 g/24 hrs and resume when proteinuria is < 2 g/24 hours<br><br>For 2+ dipstick: may administer bevacizumab; obtain 24-hour urine prior to next bevacizumab dose<br><br>For 3+ dipstick: obtain 24-hour urine prior to bevacizumab administration
Grade 3	Urinary protein >3.5 g/24 hrs	Suspend bevacizumab. Resume when proteinuria is < 2 g/24 hrs, as determined by 24-hrs urine collection <2.0 g.
Nephrotic syndrome		Discontinue bevacizumab.

6.1.2.4 *Infusion-associated reactions (not allergic)*

In case of an infusion-related reaction during the first cycle (during the 90-minute infusion or up to 24 hours later), the next infusion must be administered over at least 120 minutes.

If the 120 minute infusion is well tolerated, the next infusion and all subsequent infusions may be delivered over 120 minutes.

If any infusion-related reaction occur during the second cycle (during the 60 minute infusion or up to 24 hours later), the next infusion must be administered over 90 minutes. If the 90 minute infusion is well tolerated, the next infusion and all subsequent infusions may be delivered over 90 minutes.

If an infusion-related reaction occurs during a 30-minute infusion or up to 24 hours later, all subsequent infusions may be delivered over 60 minutes or longer.

6.1.2.5 *Surgical procedures and wound healing complications*

Bevacizumab therapy should be withheld for an interval of at least four weeks (28 days) before conducting elective surgery. In the case of unplanned surgical procedures, bevacizumab should be stopped as soon as the indication for surgery is identified. Emergency surgery should be performed as appropriate without delay after a careful risk benefit assessment.

Bevacizumab therapy should be restarted ≥28 days following major surgery. In patients who experience wound healing complications during bevacizumab treatment, bevacizumab should be withheld until the wound is fully healed.

Continuation of study treatment in patients who have had bevacizumab therapy delayed for more than 2 treatment cycles due to surgical procedures or wound healing must be carefully evaluated and eventually discussed with study coordinators.

6.1.2.6 *Thrombosis/embolism*

Arterial thromboembolism

If a patient experiences any grade of arterial thromboembolism during the study treatment period, bevacizumab should be discontinued permanently.

Venous thromboembolism

Patients experiencing a grade 4 thrombosis must discontinue-bevacizumab permanently.

If a patient experiences a grade 3 venous thromboembolism, bevacizumab must be withheld for 3 weeks. Bevacizumab may be resumed during the period of therapeutic-dose anticoagulant therapy.

Asymptomatic venous emboli and thromboses detected during the study as an incidental finding on routine CT scans should be considered on a case by case by the investigator whether the bevacizumab may be continued or not for a patient. Note that pulmonary emboli are considered as venous in origin.

6.1.2.7 *Hemorrhage*

If grade 3 or 4 bleeding of any kind occurs during the study treatment period bevacizumab should be permanently discontinued.

Dose modifications for the selected chemotherapy should be made according to local practice guidelines.

6.1.2.8 *Reversible Posterior Leucoencephalopathy Syndrome (RPLS)*

There have been rare reports of patients treated with bevacizumab that develop signs and symptoms consistent with Reversible Posterior Leucoencephalopathy Syndrome (RPLS), a rare neurological disorder, which can present with following signs and symptoms among others: seizures, headache, confusion, visual disturbance or cortical blindness, with or without associated hypertension. Brain imaging confirms the diagnosis of RPLS. Bevacizumab treatment should be discontinued in patients who develop signs/symptoms consistent with RPLS and the specific symptoms should be appropriately treated, including control of hypertension.

6.1.3 Formulation, Packaging and Labelling

Bevacizumab is supplied as a clear to slightly opalescent, colorless to pale brown, sterile liquid for intravenous infusion in single-use vials which are preservative-free. Bevacizumab will be supplied in 5 mL glass vials with a 4 mL fill (100 mg, 25 mg/mL) and/or in 20 mL glass vials with a 16 mL fill (400 mg, 25 mg/mL).

Upon receipt of the study drug, vials are to be refrigerated at 2°C-8°C (36°F-46°F) and should remain refrigerated until just prior to use. Do not freeze. Do not shake. Protect from light. Vials should not be used after the re-test date shown on the pack.

The labelling of bevacizumab will be in accordance with all local legal requirements and conducted according to Good Manufacturing Practice. (See Appendix 1 for labels used in this trial).

6.2 Carboplatin

Use normally available commercial stock in keeping with the normal practice of the institution.

There are no special accountability arrangements for carboplatin.

Reconstitute carboplatin AUC in 250ml of 5% dextrose, or according to the standard practice of the institution.

Give over 30-60 minutes (depending on local institutional practice).

6.2.1 Carboplatin dose

The carboplatin dose should be calculated according to the Calvert formula as follows:

Carboplatin dose = Target AUC (GFR + 25).

For the purpose of this protocol the GFR is considered equivalent to the creatinine clearance. The exact dose of carboplatin therefore depends on the GFR calculated by using the Cockroft-Gault formula, or the GFR measured by 24 hour urine collection.

Dose capping of carboplatin may be carried out according to local institutional protocols.

If it becomes necessary to discontinue carboplatin treatment due to hypersensitivity, patients can remain on paclitaxel and bevacizumab, switch carboplatin to cisplatin) and continue paclitaxel and bevacizumab, or continue bevacizumab-alone

6.2.1.1 GFR limitations

Patients who have had complicated or prolonged post operative recovery and who have been maintained on prolonged i.v. fluids with poor nutrition will have a falsely low serum creatinine.

Formulae such as the Cockroft-Gault formula are inaccurate at the extremes of age and weight. The calculated GFR may be falsely high in obese young women and falsely low in thin elderly women. In this case and in case of serum creatinine below the normal range the measured GFR should be take into account in Carboplatin dose definition.

It is assumed that clinicians entering patients into this protocol will be aware of these issues and the clinical judgment of an experienced clinician should be applied to the calculation of the carboplatin dose.

6.3 Paclitaxel

Use normally available commercial stock in keeping with the usual practice of the institution. There are no special accountability arrangements for paclitaxel. Reconstitute and administer via a non-PVC giving set and connectors.

6.3.1 Paclitaxel dose

Reconstitute paclitaxel 175 mg/m^2 in 250 or 500 ml of normal saline or 5% dextrose according to the standard practice of the institution. Administer over 3 hours via a rate-controlling device. Monitor closely for allergic reactions and cardiac arrhythmias as per local institution guidelines.

If it becomes necessary to discontinue paclitaxel treatment due to other toxicities, patients can remain on carboplatin and bevacizumab, or bevacizumab-alone (as described in the study design).

6.4 DOSE MODIFICATIONS AND DELAYS OF PACLITAXEL AND CARBOPLATIN

6.4.1 Minimum requirements for retreatment

- The minimum conditions for retreatment at next scheduled cycle are:
  - Absolute Neutrophil Count (ANC) ≥ 1500/mmc
  - Platelets ≥ 100.000/mmc
  - no organ toxicity grade ≥ 2

If these minimum conditions are not met, the cycle will be postponed for 7 days

6.4.2 Dose modifications and delays of carboplatin and paclitaxel

Dose modification and/or delay of paclitaxel and carboplatin have to be managed according to standard clinical practice in participating centres. The following tables reports guidelines that can be followed:

- In case of ANC < 500/mm3 or platelets < 50000/mm3 for a period of more than 7 days, the doses of all the drugs, in both the standard and the experimental arms, will be reduced by 20%.
- In case of creatinine clearance < 60 ml, the dose of carboplatin will be reduced according Table 5.
- For any G3 non-hematologic toxicity (except nausea or vomiting) the treatment should be withheld until symptoms resolve to ≤ G1. If the G3 event persists for ≥3 weeks, or recurs, then the case should be discussed with the Medical Coordinator.
- In case of peripheral neuropathy, see table 4.

Table 4. Dose modifications in case of peripheral neuropathy

Toxicity	Dose adjustments for paclitaxel
**Grade 0**	None necessary
**Grade 1**	Continue at same dose unless the patient has previously experienced grade 3-4 toxicity. In the case patient has already had grade 3-4 toxicity, hold therapy for up to 2 weeks and reduce dose by 25%.
**Grade 2**	Delay therapy up to 2 weeks or until toxicity is grade 0-1 and reduce dose by 25%. If after 2 weeks toxicity persists at grade 2, discontinue paclitaxel.
**Grade 3**	Discontinue paclitaxel.
**Grade 4**	

- Dose levels are adjusted individually for each drug.
- Up to 2 dose-reductions are acceptable for paclitaxel and carboplatin in cases of severe hematologic toxicity.
- No dose re-escalations are permitted following dose-reduction for toxicity.

6.4.2.1 *Hypersensitivity to paclitaxel and carboplatin*

Prior to paclitaxel administration, hypersensitivity prophylaxis should be implemented according to local institutional guidelines.

Close monitoring of hypersensitivity reactions to both paclitaxel and carboplatin should be performed. Hypersensitivity reactions should be managed as per local institution practice. The decision to retreat (rechallenge) a patient who experienced a hypersensitivity is up to the local investigator based on the reaction features, the personal experience and the local policies. In case of hypersensitivity to carboplatin, cisplatin can be used as alternative, since it demonstrated a reduced probability of reaction although still present. Cisplatin (75 mg/m2) would be administered i.v. over 30 minutes every 3 weeks. For patients who experience at least G3 non-hematological or G4 hematological toxicity with the cisplatin, the dose can be reduced by 25%.

6.5 Concomitant Medications

All non-cancer treatments that the responsible physician feels are appropriate are allowed in the trial.

Patients should receive full supportive care during and after the administration of bevacizumab with or without chemotherapy. This includes transfusion of blood and blood products and/or the use of erythropoietin as clinically indicated, antibiotics for infective complications and anti-hypertensive for the management of hypertension. Anaphylaxis precautions should be observed during administration of bevacizumab as per local practice. Systematic premedication to the paclitaxel administration should be given as per local practice.

Treatment with experimental concomitant, systemic anti-tumour agents or other concurrent investigational agents of any type is not allowed in this trial before protocol defined disease progression. The patient may only be entered into another therapeutic clinical trial after documented protocol defined disease progression or withdrawal from this trial.

The use of prophylactic parenteral anticoagulants is permitted as long as the INR or aPTT is within therapeutic limits (according to the medical standard in the institution) and the patient has been on a stable dose of anticoagulants for at least two weeks at the time of Day 1, Cycle 1. INR/aPTT for patients on (prophylactic) anticoagulation therapy should be checked at least before the start of every treatment cycle.

Due to a possible risk of bleeding during treatment with bevacizumab, patients should not take more than 325 mg of aspirin daily (or more than 75 mg of clopidogrel daily) at least until discontinuation of bevacizumab therapy.

All the concomitant medications should be indicated by the Investigator in the eCRF, with particular attention to the use of antidiabetics and anticoagulants.

6.6 Criteria for Premature Withdrawal

Patients have the right to withdraw from the study at any time for any reason. The investigator also has the right to withdraw patients from the study in the event of intercurrent illness, adverse events, or treatment failure after a prescribed procedure, protocol violation, cure, administrative reasons or for other reasons. An excessive rate of withdrawals could not allow to draw conclusions; therefore, unnecessary withdrawal of patients should be avoided.

Should a patient decide to withdraw, all efforts will be made to complete and report the observations as thoroughly as possible.

The investigator should contact the patient or a responsible relative by telephone or through a personal visit to establish as completely as possible the reason for the withdrawal. A complete final evaluation at the time of the patient’s withdrawal should be made with an explanation of why the patient is withdrawing from the study. If the reason for removal of a patient from the study is an adverse event the principal specific event will be recorded on the eCRF. If the AE is deemed serious, it should be recorded on the electronic SAE pages of the eCRF.

In the case that the patient decides to prematurely discontinue study treatment [“refuses treatment”], she should be asked if she can still be contacted for further information. The outcome of that discussion should be documented in both the medical records and in the eCRF.

6.6.1 Withdrawal of patients from the biomarker programme

Patients who gave consent to provide specimens have the right to withdraw their specimen from the study at any time for any reason.

7. ASSESSMENTS AND PROCEDURES DURING THE STUDY TREATMENT

7.1 Screening Examination and Eligibility Screening Form

All patients must provide written, informed consent before any study-specific assessments or procedures are performed including sampling for biomarker assessments.

Screening examinations should be performed between 1 and 28 days before the first day of treatment (unless the procedures have already been conducted during this time period as part of the patient's routine clinical care) (see Table 5).

All the consenting patients should be registered even those who result not eligible because of missing/having one or more inclusion/exclusion criteria, in order to allow the description of the generalizability of the study data. The electronic Registration Form will allow the collection of data on each single inclusion/exclusion item.

7.2 Procedures for Enrolment of Eligible Patients

Once a patient has fulfilled the entry criteria, she will be assigned a unique patient identification number when enrolled into the study. A patient enrolment list must be maintained by the investigator.

The investigator or designee will use the eCRF to enter the patient into the study.

The patient numbers will be allocated sequentially in the order in which the patients are enrolled.

A Patient Enrolment and Identification Code List must be maintained by the investigator.

7.3 Clinical Assessments and Procedures

All assessments will be scheduled as indicated in Table 5. Additional assessments may be performed as clinically indicated.

7.3.1 Tumour response criteria

Response will be evaluated according to the RECIST v 1.1 (Response Evaluation Criteria In Solid Tumours) criteria[55] summarized below.

If a serological progression of disease is suspected based on a CA-125 significant increase a radiological re-evaluation has to be performed. No change in the therapy programme will be based on Ca125 changes alone.

Evaluation of response by RECIST criteria should be performed prior (max 4 weeks before) to Day 1, Cycle 1, at the end of Cycles 3 and 6, then every 3 cycles (+/- 2 weeks of the scheduled visit) while the patient is receiving bevacizumab maintenance, and then at cessation of bevacizumab. Following cessation of bevacizumab, tumour assessment will

be performed every 3 months until disease progression. Any response that is detected will need to be confirmed by a follow-up scan at least 4 weeks later. If there is suspicion of disease progression based on clinical or laboratory findings before the next scheduled assessment, an unscheduled assessment should be performed to confirm disease progression by repeated scan.

7.3.2 Measurability of tumour lesions

At the baseline evaluation, the lesions will be defined as follows:

A. **measurable**:	Lesions which can be accurately measured on at least **one dimension** (the longest diameter must be recorded) and found to be ≥ 10 mm by CT scan and MRI or ≥ 20mm by chest X-ray.
A. **non-measurable**:	• leptomeningeal disease, ascites, pleural or pericardial effusion, inflammatory breast disease, lymphangitic involvement of skin or lung, abdominal masses/abdominal organomegaly identified by physical exam that is not measurable by reproducible imaging techniques.<br>• Bone and cystic lesions removed.<br>• Organomegaly added.

All measurements must be made using a centimetre rule or gauge.

All the baseline evaluations must be performed as close to the treatment start date as possible, and in all cases no more than 4 weeks earlier. If there is only one measurable lesion, the neoplastic nature of this lesion should be confirmed by cytologic or histologic examination.

7.3.3 Identification of "Target" and "Non-Target" lesions

All measurable lesions, up to a maximum of 2 lesions per organ and a total of 5 lesions, representing all the organs affected, are defined **target lesions** and recorded and

measured during the baseline evaluation. The target lesions should be selected on the basis of their size (the ones with the longest diameter) and the possibility of obtaining repeated measurements (through image diagnostics or clinical examination). The sum of the largest baseline diameters of all the target lesions is used as reference for defining the objective response.

All the other lesions are defined as **non target lesions** and must be recorded in the baseline evaluation. Measurement of these lesions is not required, but the presence or absence of each of them must be verified during the follow-up, at the scheduled restaging times.

7.3.4 Evaluation of target lesion response

Complete response (CR):	Disappearance of all the lesions (including the reduction to <10 mm in the shortest diameter of any pathological lymph node)
Partial Response (PR):	reduction of at least 30% in the sum of the largest diameters of the target lesions, compared to the baseline evaluation.
Progressive Disease (PD):	Target Lesions: > 20% increase in the SLD taking as reference the smallest SLD recorded since the treatment started (nadir) and minimum 5 mm increase over the nadir<br>• When sum becomes very small, increases within measurement error (2-3 mm) can lead to 20% increase
Stable disease (SD):	a reduction not sufficient to be defined as PR, compared to the baseline evaluation, or an increase not sufficient to be defined as PD, compared to the lowest sum of the largest diameters recorded since the start of treatment.

7.3.5 Evaluation of non target lesion response

Complete Response (CR):	Disappearance of all non target lesions
Non-CR/ Non-PD:	Persistence of one or more non-target lesions
Progressive Disease (PD):	Appearance of one or more new lesions or indisputable, clear progression of an existing non target lesion.

7.3.6 Evaluation of best overall response according to RECIST

The best overall response is the best response recorded from the start of treatment to the progression of the illness or a relapse (taking the lowest measurement recorded after the start of treatment as reference for the progression).

Target Lesions	Non-Target Lesions	New Lesions	**RECIST Response**
CR	CR	No	**CR**
CR	non-CR/non-PD	No	**PR**
PR	CR or non-CR/non-PD	No	**PR**
SD	CR or non-CR/non-PD	No	**SD**
PD	Any	No	**PD**
Any	PD	No	**PD**
Any	Any	Yes	**PD**

An overall deterioration in the state of health leading to a suspension of the treatment without evidence of progression of the disease will be defined as a "symptomatic worsening".

Patients who suspend treatment due to symptomatic worsening will be considered as non-responding.

7.3.7 Clinical safety assessments

At screening, patients will undergo a complete physical examination (including observable tumour measurements), measurement of vital signs (height, weight, blood pressure and body temperature), and laboratory safety assessments according to local standards and recording of all AEs grades 1-5 in the electronic CRF will be performed by the investigator. All the above assessments, except height, will be recorded at each visit until the follow-up visit 30 days after cessation of bevacizumab.

The National Cancer Institute's Cancer Toxicity Criteria for Adverse Events (NCI-CTCAE) version 4.03 will be used to grade the clinical safety of the treatment in this study. Patients will be assessed for grades 1 to 5 adverse events, including SAEs, at each clinical visit and as necessary throughout the study. All AEs will be recorded into the eCRF pages.

Safety assessments in line with local standard of care or those that are symptom-directed should be undertaken at the discretion of the treating physician.

A standard 12-lead ECG will be performed within 3 days prior to Day 1, Cycle 1 and repeated during the study as clinically indicated.

Within 30 days after the last study drug administration, patients will undergo a safety follow-up assessment, including general physical examination, measurement of vital signs, ECOG PS, laboratory assessments and AE follow-up.

Blood pressure measurement will be undertaken prior and at the end of each bevacizumab infusion; blood pressure level will be collected in the eCRF.

Table 5 – Summary table of assessment

	Screening	Combination Treatment period<br>1 Cycle = 3 weeks						Maintenance Treatment<br>1 Cycle = 3 weeks	Follow-up after bevacizumab cessation (until disease progression)	
**Cycle**	**Days -28 to -1**	**Cycle 1**	**Cycle 2**	**Cycle 3**	**Cycle 4**	**Cycle 5**	**Cycle 6**	**Cycles 9-22**	**Day 30**	**...every 3 months**
**Chemotherapy**		X	X	X	X	X	X			
**Bevacizumab**		X	X	X	X	X	X	at each cycle		
**Informed Consent**	X									
**Demographics & Concurrent Illness**	X									
**Medical History**	X									

**Haematology/ Coagulation**	X	X	X	X	X	X	X	at each cy
**Blood Chemistry**	X	X	X	X	X	X	X	at each cy

**Tumour sampling**	X	see Table 7							
**Blood Plasma**	X	see Table 7							
**DNA (genetics)**	X	see Table 7							
**Tumour Assessments**	X[3]			X[3]			X[3]	every	

Unità Sperimentazioni Cliniche
Istituto Nazionale dei Tumori di Napoli
Tel 0815903571 - fax 0817702938
e-mail: usc-segreteria@istitutotumori.na.it
www.usc-intnapoli.net

1. Pregnancy test is not required for patients without childbearing potential

2. Haematology to be performed on a weekly basis.

3. During the period of treatment the same of baseline imaging technique must be used at each

4. Abdomen/pelvis US every three cycles and Total Body CT or MRI scan every 6 cycles. Chest

Unità Sperimentazioni Cliniche
Istituto Nazionale dei Tumori di Napoli

Tel 0815903571 - fax 0817702938
e-mail: usc-segreteria@istitutotumori.na.it
www.usc-intnapoli.net

8. BIOMARKER SAMPLES AND ASSESSMENTS

In this trial the collection of both archival tissue and plasma/blood at several time pointsfrom consenting patients is mandate .

In Table 6 the timing for each sample collection is depicted.

Biomarker tests will be only performed after approval of Independent Ethical Committees. Eventual biomarkers not included in the present protocol will require a formal amendment, including the request of a specific informed consent to alive patients. All specimens will be destroyed no later than 15 years after the final freeze of the respective clinical database.

In Appendix 2 all the details regarding the laboratories where the biomarkers will be assessed and further details on biomarkers.

Table 6 – Timing of samples' collection

Sample type	Baseline	Chemotherapy Completion	PD
10 ml blood for plasma	X	X	X
5 ml blood for CEC/CEP	X	X	X
5 ml blood for DNA analyses	X		
Archival tissue	X*	at any subsequent surgical operation, if possible	

*Tissue from primary surgery or biopsy is required.

8.1.1 Specimen Types

For sampling procedures, storage conditions and shipment instructions see study Appendix 2.

Plasma assays

10 ml blood sample will be taken at each time point, centrifuged in EDTA, processed to obtain 5 ml plasma collected and stored at -20°C or (If possible) at -80°C. Plasma will be centralized and separated in 200 µl samples, in 25 aliquots, available for soluble biomarkers, proteins and miRNA.

Circulating cells

10 ml of blood will be collected at each time point and shipped to Istituto Europeo di Oncologia in Milan for circulating cells assessment and DNA extraction.

Tumour tissue sampling

Paraffin embedded histology samples will be collected from primary surgery and if possible at any subsequent surgical operation.

Formalin-fixed tumour tissue embedded in paraffin blocks (or parts of tumour blocks) with minimal necrosis will be collected along with one haematoxylin/eosin slide. These tissues may be from the primary tumour, or a metastatic site (or site of local recurrence or advancement) if the primary tumour is unavailable, or if possible from both primary tumour and a metastatic site. Tumour tissue blocks will be used to set up a tissue microarray (TMA) for IHC (ImmunoHistoChemistry) analysis and for the extraction of DNA and RNA. All the blocks will be centralized and processed at Istituto Nazionale Tumori di Napoli.

Following this process, the remaining part of the tumour block will be returned to the institution. If only part of the available tumour block was supplied for analysis, any remaining tumour material/blocks will be kept at National Cancer Institute of Naples.

9. ADVERSE EVENTS REPORTING

9.1 Definitions

An **adverse event** is any untoward medical occurrence in a patient or clinical trial subject administered a medicinal product and which does not necessarily have a causal relationship with this treatment.

An **adverse reaction** is an untoward and unintended response to an investigational medicinal product related to any dose administered, judged by either the investigator or the promoter.

An **unexpected adverse reaction** (UAR) is an adverse reaction, the nature or severity of which is not consistent with the applicable product information (e.g. investigator's brochure for an unauthorized investigational product or summary of product characteristics for an authorized product).

A **serious adverse event (SAE)** is untoward medical occurrence or effect at any dose that results in comprising or resulting in death, risk of death, permanent disability, hospitalisation or prolongation of existing hospitalization or need for urgent medical treatment. Further, any unexpected changes in relation to the toxicity profile of the drugs used of grade ≥ 3, as well as adverse event(s) which, although not falling within this definition, are considered unexpected and serious by the Investigator should be reported.

A **Suspected Unexpected Serious Adverse Reaction (SUSAR)** is an unexpected adverse reaction judged serious by the Investigator and/or Sponsor, that is not consistent, either in nature or in severity, with the applicable product information.

9.2 Collection and reporting of adverse events

All adverse events have to be reported in the toxicity case report form graded according to the corresponding CTCAE term (Version 4.03).

9.3 Collection and reporting of serious adverse events (SAE)

All serious adverse events occurring during treatment and in the 30 days after the interruption of treatment must be recorded and reported using the **serious adverse event report form** (SAE form - see enclosures).

The Investigator must immediately report to the sponsor all serious adverse events. The report should be made using the SAE form online or by sending the paper copy by fax (+390817702938) to the coordinating office immediately and not exceeding 24 hours following knowledge of the SAE.

All SAE must be also reported in the toxicity case report form within the corresponding CTCAE term.

9.4 Causality assessment between treatment and event

The following criteria will be used for causality assessment:

Term	Description
CERTAIN	A clinical event, including laboratory test abnormality, occurring in a plausible time relationship to drug administration, and which cannot be explained by concurrent disease or other drugs or chemicals. The response to withdrawal of the drug (dechallenge) should be clinically plausible. The event must be definitive pharmacologically or phenomenologically, using a satisfactory rechallenge procedure if necessary.
PROBABLE/ LIKELY	A clinical event, including laboratory test abnormality, with a reasonable time sequence to administration of the drug, unlikely to be attributed to concurrent disease or other drugs or chemicals, and which follows a clinically reasonable response on withdrawal (dechallenge). Rechallenge information is not required to fulfil this definition.
POSSIBLE	A clinical event, including laboratory test abnormality, with a reasonable time sequence to administration of the drug, but which could also be explained by concurrent disease or other drugs or chemicals. Information on drug withdrawal may be lacking or unclear.
UNLIKELY	A clinical event, including laboratory test abnormality, with a temporal relationship to drug administration which makes a causal relationship improbable, and in which other drugs, chemicals or underlying disease provide plausible explanations.

NOT RELATED	There is no causal relationship between the treatment and the event
CONDITIONAL/ UNCLASSIFIED	A clinical event, including laboratory test abnormality, reported as an adverse reaction, about which more data is essential for a proper assessment or the additional data are under examination.
UNASSESSIBLE/ UNCLASSIFIABLE	A report suggesting an adverse reaction which cannot be judged because information is insufficient or contradictory, and which cannot be supplemented or verified.

9.5 Procedures for safety reporting

NCI Naples will review all adverse and serious adverse events reported in the Study and issue queries directly to the Investigator reporting the event.

NCI Naples will determine if event qualifies as a SUSAR.

NCI Naples will report all SUSARs to the national regulatory authority "*Agenzia Italiana del Farmaco*" (AIFA), to all Participating Investigators and to all Ethical Committees of Participating Centres within the timelines of the article 17 of the European Directive 2001/20/EC.

NCI Naples will provide an annual safety report, including all Serious Adverse Events occurring in the Study, to all Participating Investigators and to Ethical Committees of Participating Centres.

Reporting flow for SAEs and SUSARs

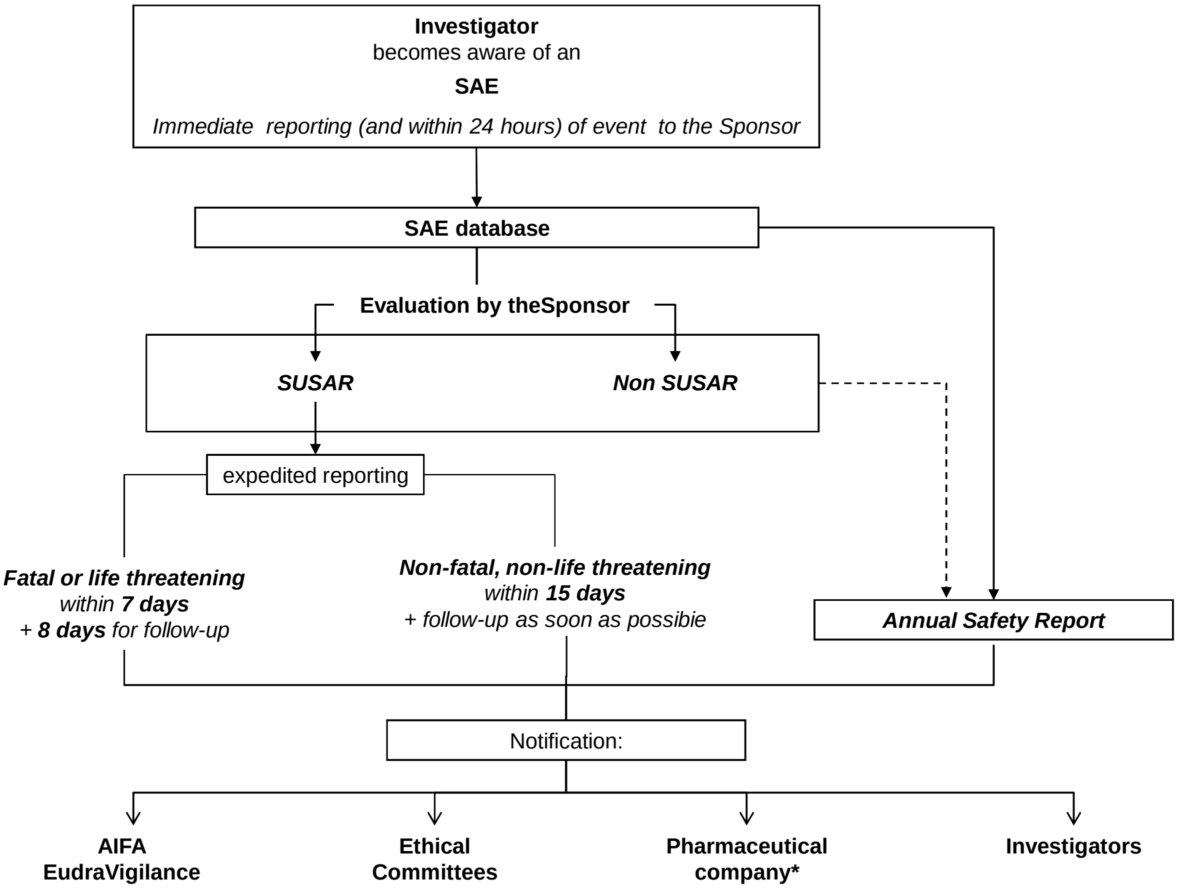

* According to trial specific agreements

10. STATISTICAL ANALYSIS

Progression Free Survival (PFS) is defined as the time elapsing from the inclusion into the study to the first occurrence of either death for any cause or disease progression. Overall survival (OAS) is defined as the time elapsing from the inclusion into the study and death for any cause. This study is exploratory in nature and tries to assess many biomarkers with different prevalence and effect on prognosis. Correlation between biomarkers is unknown, and no formal rule for adjustment for test multiplicity is defined 'a priori'. As a minimum conservative approach a significance level of 0.01 is set. Descriptive statistics will be used to describe the patient population, compliance and safety. Kaplan-Meier method will be applied to draw PFS and OAS curves. Pairwise correlation between biomarkers will also be assessed.

Potentially predictive biomarkers at baseline will be mainly assessed by multivariable proportional hazard models with age, stage, histology, PS, residual disease and centre as clinical covariates.

Dynamic biomarkers and variables whose change during or after treatment could be associated to prognosis will be entered as time-dependent covariates in multivariable Cox's model with the same clinical covariates as above.

A detailed statistical description of the methods that will be applied for each potentially predictive factor will be provided separately.

11. QUALITY ASSURANCE AND MONITORING

The procedures set out in this study protocol are designed to ensure that the promoter and the Investigators abide by the principles of the Good Clinical Practice guidelines of the International Conference on Harmonization (ICH) and the Declaration of Helsinki in the conduct, evaluation and documentation of this study. The study will be carried out adhering to local legal requirements and the applicable national law, whichever represents the greater protection for the individual. In principle, this protocol plans only centralized monitoring activities, with peripheral auditing visits planned in case of need.

12. DATA COLLECTION PROCEDURES

Patient registration and data collection are centralized at the Clinical Trials Unit of the National Cancer Institute of Naples. Patient registration is web-based (http://www.usc-intnapoli.net) or by telephone. Data collection is electronic through above website (http://www.usc-intnapoli.net), or by paper CRF transmitted by fax to +39 081-7702938, **immediately after completion.**Contacts for registration and randomization: **see contacts page**

13. ADMINISTRATIVE ASPECTS

The MITO-16/MANGO-OV2 study is a non-profit investigator initiated trial not sponsored by the pharmaceutical companies which produce the drugs used in the trial.

In this trial, all the drugs (including bevacizumab) are used in-label; however, at the time of study proposal, bevacizumab is not yet authorized for reimbursement by the public health system in Italy. Therefore it will be provided at no cost to the centres by the manufacturer, for all the study duration.

Considering that collection of blood samples for specific scientific purposes is planned, the promoter (National Cancer Institute of Naples) will provide an insurance policy to cover possible damages caused to patients participating in the trial. The policy will cover all participating centres in Italy.

An agreement between the Coordinating Centre and each participating centre will be stipulated.

Study protocol, patient information and informed consent will be submitted to the appropriate Ethical Committees for approval. At each centre, the study will only be started after being approved by the institutional Ethical Committee. The promoter will inform the Ethical Committees about any changes in the study protocol which could interfere with the patient’s safety. Furthermore, the institutional review board will be informed of the planned or premature end of the study.

14. COORDINATING CENTER CONTACTS

Principal Investigators

MITO:

Dr. Sandro Pignata, MD, PhD
Division of Medical Oncology – Uro-Gynecology Department
Tel: +39 081-5903637
Fax: +39 081-5903861
Email: s.pignata@istitutotumori.na.it
National Cancer Institute Naples

MaNGO:

Prof. Nicoletta Colombo, MD
Unit of Medical Gynecologic Oncology –
Tel: +39 02-57489543
Fax: +39 02-94379222
Email: nicoletta.colombo@ieo.it
European Institute of Oncology
University of Milan-Bicocca

Coordinating centre
Clinical Trials Unit:
National Cancer Institute Naples
Dr Francesco Perrone, MD, PhD
Dr. Gennaro Daniele, MD, PhD
Dr. Jane Bryce, RN
Tel: +39 081 - 5903571
Fax: +39 081 - 7702938
E-mail:francesco.perrone@usc-intnapoli.net;
gennaro.daniele@usc-intnapoli.net;
jane.bryce@usc-intnapoli.net

Coordinating centre for MaNGO
Gynaecology-Oncology Unit:
Mario Negri Research Institute
Dr Roldano Fossati
Tel: +39 02 390 14 467
Fax: +39 02 33 200 231
E-mail: roldano.fossati@marionegri.it

Study Statisticians

- Prof. Ciro Gallo

Department of Medical Statistics
Second University of Naples
E-mail: ciro.gallo@unina2.it

- Dr Irene Floriani

Clinical Trials Lab
Mario Negri Research Institute
E-mail: irene.floriani@marionegri.it

Contacts for registration and randomization:
Cristiana De Luca, Giuliana Canzanella, Federika Crudele, Manuela Florio, Giovanni de Matteis
Clinical Trials Unit
National Cancer Institute Naples
http://www.usc-intnapoli.net
e-mail: datamanager@ usc-intnapoli.net
tel: + 39 081-5903571
fax: + 39 081-7702938
Monday through Friday: 9.30 – 15.30

15. REFERENCES

1. Siegel R, Naishadham D, Jemal A: Cancer statistics, 2012. CA Cancer J Clin 62:10-29, 2012
2. Chan JK, Cheung MK, Husain A, et al: Patterns and progress in ovarian cancer over 14 years. Obstet Gynecol 108:521-8, 2006
3. Engel J, Eckel R, Schubert-Fritschle G, et al: Moderate progress for ovarian cancer in the last 20 years: prolongation of survival, but no improvement in the cure rate. Eur J Cancer 38:2435-45, 2002
4. Bookman MA: Developmental chemotherapy and management of recurrent ovarian cancer. J Clin Oncol 21:149s-167s, 2003
5. Harries M, Gore M: Part I: chemotherapy for epithelial ovarian cancer-treatment at first diagnosis. Lancet Oncol 3:529-36, 2002
6. International Collaborative Ovarian Neoplasm G: Paclitaxel plus carboplatin versus standard chemotherapy with either single-agent carboplatin or cyclophosphamide, doxorubicin, and cisplatin in women with ovarian cancer: the ICON3 randomised trial. Lancet 360:505-15, 2002
7. Armstrong DK, Bundy B, Wenzel L, et al: Intraperitoneal cisplatin and paclitaxel in ovarian cancer. N Engl J Med 354:34-43, 2006
8. McGuire WP, Hoskins WJ, Brady MF, et al: Cyclophosphamide and cisplatin compared with paclitaxel and cisplatin in patients with stage III and stage IV ovarian cancer. N Engl J Med 334:1-6, 1996
9. Muggia FM, Braly PS, Brady MF, et al: Phase III randomized study of cisplatin versus paclitaxel versus cisplatin and paclitaxel in patients with suboptimal stage III or IV ovarian cancer: a gynecologic oncology group study. J Clin Oncol 18:106-15, 2000
10. Piccart MJ, Bertelsen K, James K, et al: Randomized intergroup trial of cisplatin-paclitaxel versus cisplatin-cyclophosphamide in women with advanced epithelial ovarian cancer: three-year results. J Natl Cancer Inst 92:699-708, 2000
11. Spannuth WA, Sood AK, Coleman RL: Angiogenesis as a strategic target for ovarian cancer therapy. Nat Clin Pract Oncol 5:194-204, 2008
12. du Bois A, Quinn M, Thigpen T, et al: 2004 consensus statements on the management of ovarian cancer: final document of the 3rd International Gynecologic Cancer Intergroup Ovarian Cancer Consensus Conference (GCIG OCCC 2004). Annals of Oncology 16:viii7-viii12, 2005
13. Presta LG, Chen H, O'Connor SJ, et al: Humanization of an anti-vascular endothelial growth factor monoclonal antibody for the therapy of solid tumors and other disorders. Cancer Res 57:4593-9, 1997
14. Jain RK: Normalizing tumor vasculature with anti-angiogenic therapy: a new paradigm for combination therapy. Nat Med 7:987-9, 2001
15. Sweeney CJ, Miller KD, Sissons SE, et al: The antiangiogenic property of docetaxel is synergistic with a recombinant humanized monoclonal antibody against vascular endothelial growth factor or 2-methoxyestradiol but antagonized by endothelial growth factors. Cancer Res 61:3369-72, 2001
16. Borgstrom P, Gold DP, Hillan KJ, et al: Importance of VEGF for breast cancer angiogenesis in vivo: implications from intravital microscopy of combination

treatments with an anti-VEGF neutralizing monoclonal antibody and doxorubicin. Anticancer Res 19:4203-14, 1999

17. Yuan F, Chen Y, Dellian M, et al: Time-dependent vascular regression and permeability changes in established human tumor xenografts induced by an anti-vascular endothelial growth factor/vascular permeability factor antibody. Proc Natl Acad Sci U S A 93:14765-70, 1996
18. Kanai T, Konno H, Tanaka T, et al: Anti-tumor and anti-metastatic effects of human-vascular-endothelial-growth-factor-neutralizing antibody on human colon and gastric carcinoma xenotransplanted orthotopically into nude mice. Int J Cancer 77:933-6, 1998
19. Burger RA, Brady MF, Bookman MA, et al: Incorporation of bevacizumab in the primary treatment of ovarian cancer. N Engl J Med 365:2473-83, 2011
20. Perren TJ, Swart AM, Pfisterer J, et al: A phase 3 trial of bevacizumab in ovarian cancer. N Engl J Med 365:2484-96, 2011
21. Cannistra SA, Matulonis UA, Penson RT, et al: Phase II study of bevacizumab in patients with platinum-resistant ovarian cancer or peritoneal serous cancer. J Clin Oncol 25:5180-6, 2007
22. Burger RA, Sill MW, Monk BJ, et al: Phase II trial of bevacizumab in persistent or recurrent epithelial ovarian cancer or primary peritoneal cancer: a Gynecologic Oncology Group Study. J Clin Oncol 25:5165-71, 2007
23. Nazer B, Humphreys BD, Moslehi J: Effects of novel angiogenesis inhibitors for the treatment of cancer on the cardiovascular system: focus on hypertension. Circulation 124:1687-91, 2011
24. Mourad JJ, des Guetz G, Debbabi H, et al: Blood pressure rise following angiogenesis inhibition by bevacizumab. A crucial role for microcirculation. Ann Oncol 19:927-34, 2008
25. Bono P, Elfving H, Utriainen T, et al: Hypertension and clinical benefit of bevacizumab in the treatment of advanced renal cell carcinoma. Ann Oncol 20:393-4, 2009
26. Scartozzi M, Galizia E, Chiorrini S, et al: Arterial hypertension correlates with clinical outcome in colorectal cancer patients treated with first-line bevacizumab. Ann Oncol 20:227-30, 2009
27. Schneider BP, Wang M, Radovich M, et al: Association of vascular endothelial growth factor and vascular endothelial growth factor receptor-2 genetic polymorphisms with outcome in a trial of paclitaxel compared with paclitaxel plus bevacizumab in advanced breast cancer: ECOG 2100. J Clin Oncol 26:4672-8, 2008
28. Dahlberg SE, Sandler AB, Brahmer JR, et al: Clinical course of advanced non-small-cell lung cancer patients experiencing hypertension during treatment with bevacizumab in combination with carboplatin and paclitaxel on ECOG 4599. J Clin Oncol 28:949-54, 2010
29. Rini BI, Halabi S, Rosenberg JE, et al: Phase III trial of bevacizumab plus interferon alfa versus interferon alfa monotherapy in patients with metastatic renal cell carcinoma: final results of CALGB 90206. J Clin Oncol 28:2137-43, 2010
30. Hurwitz H, Douglas PS, Middleton JP, et al: Analysis of early hypertension (HTN) and clinical outcome with bevacizumab (BV). Journal Clin. Oncol. 28, 2010

31. Jain RK, Duda DG, Willett CG, et al: Biomarkers of response and resistance to antiangiogenic therapy. Nat Rev Clin Oncol 6:327-38, 2009

32. Carrillo-de Santa Pau E, Carrillo Arias F, Caso Pelaez E, et al: Vascular endothelial growth factor (VEGF) serum levels are associated with survival in early stages of lung cancer patients. Cancer Invest 28:393-8, 2010

33. Bernaards C, Hegde P, Chen D, et al: Circulating vascular endothelial growth factor (VEGF) as a biomarker for bevacizumab-based therapy in metastatic colorectal, non-small cell lung, and renal cell cancers: Analysis of phase III studies. ASCO Meeting Abstracts 28:10519, 2010

34. Bais C, Rabe C, Wild N, et al: Comprehensive reassessment of plasma VEGFA (pVEGFA) as a candidate predictive biomarker for bevacizumab (Bv) in 13 pivotal trials (seven indications), ASCO Annual Meeting 2014, 2014

35. Miles D, Haas Sd, Dirix L, et al: Plasma biomarker analyses in the AVADO Phase III randomized study of firts-line bevacizumab + docetaxel in patients with HER2-negative metastatic breast cancer. Cancer Research Suppl 70:235, 2010

36. Van Cutsem E, Jayson G, Dilba P, et al: Analysis of blood plasma factors in the AVITA phase III randomized study of bevacizumab (bev) with gemcitabine-erlotinib (GE) in patients (pts) with metastatic pancreatic cancer (mPC). European Society for Medical Oncology 2011:abstract 803., 2011

37. Shah MA, Ohtsu A, al. e: Tumor and blood plasma biomarker analyses in the AVAGAST phase III randomized study of first-line bevacizumab + capecitabine/ cisplatin in patients with advanced gastric cancer. European Society for Medical Oncology 2010:abstract 4675., 2010

38. Van Cutsem E, de Haas S, Kang Y-K, et al: Bevacizumab in Combination With Chemotherapy as First-Line Therapy in Advanced Gastric Cancer: A Biomarker Evaluation From the AVAGAST Randomized Phase III Trial. Journal of Clinical Oncology, 2012

39. Jayson G, De Haas S, Delmar P, et al: Evaluation of plasma VEGFA as a potential predictive pan-tumour biomarker for bevacizumab. European Society for Medical Oncology 2011:abstract 804., 2011

40. Baar J, Silverman P, Lyons J, et al: A vasculature-targeting regimen of preoperative docetaxel with or without bevacizumab for locally advanced breast cancer: impact on angiogenic biomarkers. Clin Cancer Res 15:3583-90, 2009

41. Lu JF, Bruno R, Eppler S, et al: Clinical pharmacokinetics of bevacizumab in patients with solid tumors. Cancer Chemother Pharmacol 62:779-86, 2008

42. Willett CG, Duda DG, di Tomaso E, et al: Efficacy, safety, and biomarkers of neoadjuvant bevacizumab, radiation therapy, and fluorouracil in rectal cancer: a multidisciplinary phase II study. J Clin Oncol 27:3020-6, 2009

43. Loupakis F, Falcone A, Masi G, et al: Vascular endothelial growth factor levels in immunodepleted plasma of cancer patients as a possible pharmacodynamic marker for bevacizumab activity. J Clin Oncol 25:1816-8, 2007

44. Das Thakur M, Bais C, Estay I, et al: Biomarker (BM) results from MERiDiAN, a doubleblind placebo (PLA)controlled randomized phase 3 trial of 1stline paclitaxel (PAC) with or without bevacizumab (BEV) for HER2negative

metastatic breast cancer (mBC). Presented at the 38th San Antonio Breast Cancer Symposium, San Antonio, TX, 2015

45. Jubb AM, Harris AL: Biomarkers to predict the clinical efficacy of bevacizumab in cancer. Lancet Oncol 11:1172-83, 2010
46. Dowlati A, Gray R, Sandler AB, et al: Cell adhesion molecules, vascular endothelial growth factor, and basic fibroblast growth factor in patients with non-small cell lung cancer treated with chemotherapy with or without bevacizumab--an Eastern Cooperative Oncology Group Study. Clin Cancer Res 14:1407-12, 2008
47. Kerbel RS: Tumor angiogenesis. N Engl J Med 358:2039-49, 2008
48. Mancuso P, Colleoni M, Calleri A, et al: Circulating endothelial-cell kinetics and viability predict survival in breast cancer patients receiving metronomic chemotherapy. Blood 108:452-9, 2006
49. Ronzoni M, Manzoni M, Mariucci S, et al: Circulating endothelial cells and endothelial progenitors as predictive markers of clinical response to bevacizumab-based first-line treatment in advanced colorectal cancer patients. Ann Oncol 21:2382-9, 2010
50. Schneider BP, Wang M, Radovich M, et al: Association of vascular endothelial growth factor and vascular endothelial growth factor receptor-2 genetic polymorphisms with outcome in a trial of paclitaxel compared with paclitaxel plus bevacizumab in advanced breast cancer: ECOG 2100. J Clin Oncol 26:4672-8, 2008
51. Schultheis AM, Lurje G, Rhodes KE, et al: Polymorphisms and clinical outcome in recurrent ovarian cancer treated with cyclophosphamide and bevacizumab. Clin Cancer Res 14:7554-63, 2008
52. Zhang W, Dahlberg SE, Yang D, et al: genetic variants in angiogenesis pathway associated with clinical outcome in NSCLC patients (pts) treated with bevacizumab in combination with carboplatin and paclitaxel: Subset pharmacogenetic analysis of ECOG 4599. J Clin Oncol 27:abstract 8032, 2009
53. Lambrechts D, Delmar P, Buysschaert I, et al: VEGFR-1 polymorphisms as potential predictors of clinical outcome in bevacizumab-treated patients with metastatic pancreatic cancer. Eur J Cancer Suppl 7:10, 2009
54. Schoenfeld DA: Sample-size formula for the proportional-hazards regression model. Biometrics 39:499-503, 1983
55. Eisenhauer EA, Therasse P, Bogaerts J, et al: New response evaluation criteria in solid tumours: revised RECIST guideline (version 1.1). Eur J Cancer 45:228-47, 2009

16. APPENDIX 1: BEVACIZUMAB LABELING

Etichetta imballaggio esterno o confezionamento secondario:
Protocollo ML28412 – MITO16_MangoOV2_Phase IV
Sperimentatore__________
N. paziente_________ Data di somministrazione___________
1 flaconcino di bevacizumab 400 mg/16 ml (25 mg/ml)
Concentrato per soluzione per infusione ev.
Lotto n°_________ Data di scadenza_ _______
Utilizzare come prescritto nel protocollo.
Conservare a + 2℃ - 8℃. Non conge lare. Non agitare.
Tenere il flaconcino nell'imballaggio esterno.
Solo per uso sperimentale.
Restituire tutte le confezioni di farmaco vuote e i prodotti non utilizzati allo sperimentatore.
Istituto Nazionale dei Tumori IRCCS - Fondazione G. Pascale – Via Mariano Semmola – 80131 Napoli

Etichetta flaconcino o confezionamento primario:
Protocollo ML28412 – MITO16_MangoOV2_Phase IV
Sperimentatore__________
N. paziente__________ Data di somministrazione___________
1 flaconcino di bevacizumab 400 mg/16 ml (25 mg/ml)
Concentrato per soluzione per infusione ev.
Lotto n°_________ Data di scadenza_ _______
Istituto Nazionale dei Tumori IRCCS - Fondazione G. Pascale – Via Mariano Semmola – 80131 Napoli

17. APPENDIX 2: SAMPLES HANDLING AND SHIPPING

Etichetta imballaggio esterno o confezionamento secondario:
Protocollo ML28412 – MITO16_MangoOV2_Phase IV
Sperimentatore__________
N. paziente_________ Data di somministrazione___________
1 flaconcino di bevacizumab 400 mg/16 ml (25 mg/ml)
Concentrato per soluzione per infusione ev.
Lotto n°_________ Data di scadenza_ _______
Utilizzare come prescritto nel protocollo.
Conservare a + 2℃ - 8℃. Non conge lare. Non agitare.
Tenere il flaconcino nell'imballaggio esterno.
Solo per uso sperimentale.
Restituire tutte le confezioni di farmaco vuote e i prodotti non utilizzati allo sperimentatore.
Istituto Nazionale dei Tumori IRCCS - Fondazione G. Pascale – Via Mariano Semmola – 80131 Napoli

Etichetta flaconcino o confezionamento primario:
Protocollo ML28412 – MITO16_MangoOV2_Phase IV
Sperimentatore__________
N. paziente__________ Data di somministrazione___________
1 flaconcino di bevacizumab 400 mg/16 ml (25 mg/ml)
Concentrato per soluzione per infusione ev.
Lotto n°_________ Data di scadenza_ _______
Istituto Nazionale dei Tumori IRCCS - Fondazione G. Pascale – Via Mariano Semmola – 80131 Napoli

Samples for circulating plasma biomarkers (including miRNA):

1. Check the patient signature on the consent.
2. Draw 10 mls of blood into EDTA tubes with a Vacutainer® sysytem and avoiding tourniquet (in order to avoid hemolysis)
3. Gently (vigorous mixing can cause haemolysis) invert the tube 8-10 times to mix the anticoagulant with the blood
4. Within 30 minutes of blood collection, spin the blood in a refrigerated centrifuge at 4° , at 1500g for 10 minutes.
5. **Immediately** after centrifugation, carefully transfer the plasma into the labelled transfer tube using a plastic pipette **RNAse free**. Please be sure to not aspirate the interface containing the platelets.
6. Store the plasma obtained in a -70° to -80° freezer until the shipment to the collecting centre (If only -20° is available, it is acceptable)

Samples DNA markers (Pharmacogenomics)

1. Check the patient signature for DNA testing on the consent.
2. Draw 5 mls of blood into EDTA tubes.
3. Gently (vigorous mixing can cause haemolysis) invert the tube 8-10 times to mix the anticoagulant with the blood
4. Put the tube in a -20° freezer (tubes must not be put in -70° directly as they may crack)
5. Samples can be stored at -20° or can be moved to -70°0 or -80° for long term storage.

Samples for circulating cells (CEC/CEP)

1. Double-check the patient signature for DNA testing on the consent.
2. Collect 4 mls of blood in the dedicated BD Vacutainer® CPT™ Tube, prefilled with Sodium-Citrate, provided within the trial materials.
3. Gently (vigorous mixing can cause haemolysis) invert the tube 8-10 times to mix the anticoagulant with the blood, repeat this operation immediately before centrifugation if this does not occur quickly after the blood collection.
4. Within 2 hr from collection, centrifuge the tube at room temeperature for 25 min at 1,600g.
5. Transfer the mononuclear cells into the cryovial and add an equal volume and add an equal volume of freezing medium (RPMI1640 with 20% DMSO to the final concentration of 10%) to the cell suspension.

6. Perform controlled Freezing into a isopropanol bath into a -80° freezer
7. Store the samples in liquid nitrogen until the day of shipping.

18. APPENDIX 3: TRANSLATIONAL PROJECT

Description of the biomarkers investigated and of the tasks of all the research groups involved

In 2012, several research groups decided to investigate potential prognostic/potential biomarkers in ovarian cancer patients enrolled in the MITO 16/MANGO OV2/ENGOT OV17 trial. Candidate biomarkers were chosen based on available preliminary data generated by the groups and literature information.

In 2015, new evidence from literature prompted the research groups to revise the translational project and to update the proposal with new biomarkers to be evaluated on the samples collected.

The genes and genes products to be studied in MITO16 patients' samples are detailed below, along with the laboratory performing the test and sample amount needed, and comprise 8 distinct groups:

1) Proteins directly related with the angiogenetic process and the biological activity of Bevacizumab. These biomarkers will be tested in collected plasma and/or tissue samples by using different techniques including multiplex array (plasma), IHC (tissue slices from TMA or whole sample section) or dedicated ELISA assay (plasma).

2) Proteins classifiable as prognostic and/or predictive biomarkers. These biomarkers will be tested in collected plasma and/or tissue samples by using IHC (tissue slices from TMA or whole sample section) or dedicated ELISA assay (plasma).

3) Genes possibly altered and/or mutated in OC. In the last years it has been clearly demonstrated that mutation of specific genes could impact on the response to therapy of OC. For this reason we designed a panel of 140 genes that will be sequenced by NGS (Illumina), analyzing for each patient both tumor and constitutive DNA. Moreover, given the relevance of BRCA1/2 mutations in the response to platinum and possibly also to antiangiogenic compounds, we anticipate that all patients will also be sequenced for BRCA1/2 for diagnostic purposes.

4) Prognostic and/or predictive micro RNA signature. In the last 2 years, three independent MITO/MANGO groups defined the prognostic/predictive role of specific miRs in ovarian cancer. qRT-PCR and/or Digital PCR analyses will be employed to quantify the expression of selected miRs in tumor samples and to confirm their prognostic/predictive value.

5) Pharmacogenetics markers of therapy outcome. We will look at polymorphisms in genes related to drugs metabolism (e.g. CYP2C8), cellular detoxification (ABCB1) and platinum response (ERCC1, ERCC2) and in genes related to the angiogenesis (e.g. VEGF) by using an NGS approach in constitutive DNA collected at diagnosis from patients enrolled in the study.

6) Proteomic and metabolomic profiling in a differential approach of a selected patient plasma samples collected at the 4 time points will be performed using a label free Liquid

Chromathography-Mass Spectrometry (LC-MS) technology. Data analysis will allow the identification of the most relevant and validated proteins. The final selected proteins panel will be validated and will work as pharmacodynamic biomarker for drug efficacy monitoring. Data analysis will include all the biomarkers listed in the summary of the proposed biomarkers (Table of angiogenesis markers, Table of prognostic markers, Table of NGS seq gene panel).

7) Evaluation of the predictive/prognostic role of Circulating Endothelial Cells/circulating Endothelial Precursors (CEC/CEP)

8) Prognostic/predictive value of tRNA derived-small RNA (tsRNA). Recent evidence suggested that certain microRNA clusters are associated with a novel class of non-coding RNA formed during the tRNA processing (tsRNA). These molecules are shown to be expressed in many tumours and evidence supports that are linked with the regulation of some oncogenes (i.e. TCL1 in CLL). These molecules will b evaluated with a custom array of 120 tsRNA of ≥16bp.

Whenever possible, biomarker analyses will be centralized in a single laboratory with the agreement that all results generated will be available to the participants upon completion of the translational study.

T*ABLE ANGIOGENESIS MARKER*

1.1 Tissutal markers (TISSUE 4 µm)

Biomarker	Technique	Performing Lab	Sample amount
Podoplanin	IHC	PASCALE NAPOLI - LOSITO	1 WHOLE SAMPLE SECTION NO TMA
CD34	IHC	PASCALE NAPOLI - LOSITO	1 WHOLE SAMPLE SECTION NO TMA
αSMA	IHC	SPEDALI RIUNITI BRESCIA - TOGNON/BIGNOTTI	1 WHOLE SAMPLE SECTION NO TMA
TSP-1a	IHC	SPEDALI RIUNITI BRESCIA - TOGNON/BIGNOTTI	1 WHOLE SAMPLE SECTION
CXCL-9	IHC	IOV PADOVA - INDRACCOLO	1 TMA
CXCL-10	IHC	IOV PADOVA - INDRACCOLO	1 TMA
CA IX	IHC	IOV PADOVA - INDRACCOLO	1 TMA
MULTIMERIN	IHC	CRO AVIANO - BALDASSARRE	1 WHOLE SAMPLE SECTION
FGFR-4	IHC	CRO AVIANO - BALDASSARRE	1 TMA
Survivin	IHC	PASCALE NAPOLI – CALIFANO CHIAPPETTA	1 TMA
CD31	IHC	UNICATT – ROMA FERRANDINA	1 WHOLE SAMPLE SECTION NO TMA
VEGFR-1, 2	IHC	FERRANDINA	2 WHOLE SAMPLE SECTION
VEGF-B	IHC	FERRANDINA	1 TMA
IL-6	IHC	INT MILANO - MEZZANZANICA/TOMASSETTI	1 WHOLE SAMPLE SECTION
Endothelin Receptor A-B	IHC	PASCALE NAPOLI – CALIFANO CHIAPPETTA	2 TMA
Endothelin 1-3 (ET-1)	IHC	PASCALE NAPOLI – CALIFANO CHIAPPETTA	3 TMA
HIF-1α	IHC	CHIAPPETTA/CALIFANO	1 WHOLE SAMPLE SECTION
CD8	IHC	CHIAPPETTA/LOSITO	1 WHOLE SAMPLE SECTION

1.2: Plasmatic markers

Biomarker	Technique	Performing Lab	Sample amount
VEGF- D	ELISA	SPEDALI RIUNITI BRESCIA - TOGNON/BIGNOTTI	200 µl
PDGF-BB	ELISA	SPEDALI RIUNITI BRESCIA - TOGNON/BIGNOTTI	200 µl
FLT4	ELISA	SPEDALI RIUNITI BRESCIA - TOGNON/BIGNOTTI	200 µl
AGP	ELISA	SPEDALI RIUNITI BRESCIA - TOGNON/BIGNOTTI	200 µl
IL-1β	ELISA	SPEDALI RIUNITI BRESCIA - TOGNON/BIGNOTTI	200 µl
TNF-α	ELISA	SPEDALI RIUNITI BRESCIA - TOGNON/BIGNOTTI	200 µl
ANG1, 2	ELISA	SPEDALI RIUNITI BRESCIA - TOGNON/BIGNOTTI	200 µl
TIE2	ELISA	SPEDALI RIUNITI BRESCIA - TOGNON/BIGNOTTI	200 µl
PlGF	ELISA	SPEDALI RIUNITI BRESCIA - TOGNON/BIGNOTTI	200 µl
CA IX	ELISA	SPEDALI RIUNITI BRESCIA - TOGNON/BIGNOTTI	200 µl
IGF-1	ELISA	SPEDALI RIUNITI BRESCIA - TOGNON/BIGNOTTI	200 µl
HGF	ELISA	SPEDALI RIUNITI BRESCIA - TOGNON/BIGNOTTI	200 µl
MµLTIMERIN	ELISA	CRO AVIANO - BALDASSARRE	
VEGF-A, C	MULTIPLEX ELISA	tbd	
VEGF-B	ELISA	BERTOLINI	
VEGFR 3		FERRANDINA	
VEGFR 1, 2	MULTIPLEX ELISA	ERRANDINA	
FGF2	MULTIPLEX ELISA	tbd	
IL-8	MULTIPLEX ELISA	tbd	
G-CSF	MULTIPLEX ELISA	tbd	
SDF-1α	MULTIPLEX ELISA	tbd	
e-selectin	MULTIPLEX ELISA	tbd	
PDGF_C	MULTIPLEX ELISA	tbd	
ICAM-1	MULTIPLEX ELISA	tbd	

IL-6	MULTIPLEX ELISA	INT MILANO - MEZZANZANICA /BERTOLINI	200µL
PDGF		BERTOLINI	
TSP-1		MARIO NEGRI-GIAVAZZI	20 µl
MMP2		MARIO NEGRI-GIAVAZZI	20 µl

**Biomarker**	**Technique**	**Performing Lab**	**Sample amount**
**MMP9**		**MARIO NEGRI-GIAVAZZI**	**20 µl**
**TIMP1**		**MARIO NEGRI-GIAVAZZI**	**20 µl**
**TIMP2**		**MARIO NEGRI-GIAVAZZI**	**20 µl**

2) TABLE PROGNOSTIC MARKERS

2.1 Tissutal markers (TISSUE 4 µm)

Biomarker	Technique	Performing Lab	Sample amount
Ki67	IHC	SPEDALI RIUNITI BRESCIA - TOGNON/BIGNOTTI	1 WHOLE SAMPLE SECTION
TP53	IHC	SPEDALI RIUNITI BRESCIA - TOGNON/BIGNOTTI	1 WHOLE SAMPLE SECTION
AXL	IHC	INT MILANO - MEZZANZANICA/TOMASSETTI	1 TMA
p130Cas	IHC	INT MILANO - MEZZANZANICA/TOMASSETTI	1 TMA
TIMP-3	IHC	INT MILANO - MEZZANZANICA/TOMASSETTI	1 TMA
pSMAD2	IHC	CATTOLICA ROMA - ZANNONI	1 WHOLE SAMPLE SECTION
DNA-PK	IHC	IOV PADOVA - INDRACCOLO	1 TMA
GLS	IHC	IOV PADOVA - INDRACCOLO	1 TMA
CD3	IHC	IOV PADOVA - INDRACCOLO	1 WHOLE SAMPLE SECTION NO TMA
CD274	IHC	CRO AVIANO - BALDASSARRE	1 WHOLE SAMPLE SECTION
MESOTHELIN	IHC	IST GENOVA - FERRINI/FABBI	1 WHOLE SAMPLE SECTION
CXCR4	IHC	PASCALE NAPOLI - SCALA	1 TMA

CXCL12	IHC	PASCALE NAPOLI - SCALA	1 TMA
CXCR7	IHC	PASCALE NAPOLI - SCALA	1 TMA
BRCA1	IHC	PASCALE NAPOLI - LOSITO	1 WHOLE SAMPLE SECTION
MLH1	IHC	IRE Roma-CAROSI	1 TMA
MSH2	IHC	IRE Roma-CAROSI	1 TMA
RASSF1A	IHC	IRE Roma-CAROSI	1 TMA
CDKN2A	IHC	IRE Roma-CAROSI	1 TMA
PTEN	IHC	IRE Roma-CAROSI	1 TMA
PAI-1	IHC	INT MILANO - MEZZANZANICA/TOMASSETTI	1 TMA
EGFR	IHC	INT MILANO - MEZZANZANICA/TOMASSETTI	1 TMA
HOXB3/D3	IHC	PASCALE NAPOLI - LOSITO	2 TMA
EPHA/EPHAR	IHC	PASCALE NAPOLI - LOSITO	2 TMA
EPHB/EPHBR	IHC	PASCALE NAPOLI - LOSITO	2 TMA
pACC	IHC	IOV PADOVA - INDRACCOLO	1 TMA
MCT1	IHC	IOV PADOVA - INDRACCOLO	1 TMA
MCT4	IHC	IOV PADOVA - INDRACCOLO	1 TMA
ADAM17/TACE	IHC	IST GENOVA - FERRINI/FABBI	1 WHOLE SAMPLE SECTION
TRAP 1	IHC	UNINA-ESPOSITO	1 TMA
HSP90	IHC	UNINA-ESPOSITO	1 TMA
Grp78/Bip	IHC	UNINA-ESPOSITO	1 TMA
Chop	IHC	UNINA-ESPOSITO	1 TMA

2.2 Plasmatic markers

Biomarker	Technique	Performing Lab	Sample amount
MESOTHELIN	ELISA	IST GENOVA - FERRINI/FABBI	150 µl
CXCL12	ELISA	PASCALE NAPOLI - SCALA	200 µl
PAI-1	ELISA	INT MILANO – MEZZANZANICA	200 µl
sALCAM	ELISA	IST GENOVA - FERRINI/FABBI	150 µl

3) TABLE PROGNOSTIC DNA MARKERS (DNA from FFPE will be extracted with GeneRead DNA FFPE Kit cod 180134)

Genes altered/mutated/methylated in OC

Biomarker	Technique	Performing Lab	Sample amount (both from tumor and normal cells)
140 gene panel*	NGS	MARIO NEGRI D'INCALCI/MARCHINI	100 ng DNA
BRCA1/2	NGS	CATTOLICA CAPOLUONGO/SCAMBIA	100 ng DNA
Methylation: BRCA1, MLH1, MSH2, RASSF1A, PTEN, CDKN2A	Pyrosequencing System and methylation-specific MLPA	IRE ROMA CAROSI	1.2 µg DNA

4) PROGNOSTIC miR SIGNATURE (RNA from FFPE will be extracted with miRNeasy FFPE Kit cod 217504)

Performing Lab	Technique	Sample amount
D'Incalci	miR-181 (PCR)	300 ng
Mezzanzanica	miR expression profile	1500 ng
Mezzanzanica	Gene expression profile (RNAseq)	
Carosi	miR-200 family (miR-200a, -200c, -141, 214) (PCR)	500 ng
Baldassarre	4 miR signature angio-related (PCR)	300-500 ng

5) PHARMACOGENETICS MARKERS OF THERAPY OUTCOME

Biomarker	Technique	Performing Lab	Sample amount
159 gene panel**	NGS	CECCHIN/TOFFOLI	1 ml (whole blood)

6) PROTEOMIC and METABOLOMIC PROFILING

Performing Lab	Technique	Sample amount
Costi	LC-MS	Plasma 2 ml

7) CEC/CEP

Performing Lab	Sample
LAB: Bertolini	Blood for CEC/CEP

8)tsRNA

Performing Lab	Sample
LAB: Croce	RNA from FFPE

*NGS SEQ gene Panel (140 genes)

Mario Negri target sequencing panel for ovarian cancer (140 GENES)										
DNA Repair						Cell signaling			Miscellaneous	cell cycle
ATM	BER	FA/HR	MMR	NER	NHEJ					
ATR	ALKBH2	BRCA1	MLH1	DDB1	POLM	AKT1	KRAS	NOTCH1	APC	RB1
ATM	ALKBH2	BRCA2	MLH3	DDB2	NHEJ1	AKT2	BRAF	NOTCH2	ARID1A	CDH1
CHEK1	MGMT	BRIP1	MSH2	ERCC1	XRCC4	MTOR	NRAS	NOTCH3	ARID2	CDK12
CHEK2	PARP1	C17orf70	MSH3	ERCC2	XRCC5	TP53		MYC	DROSHA	CDK4
H2AFX	PARP2	c11orf30	MSH6	ERCC3	XRCC6	TP53BP1	ERBB2	NF1	DICER1	CDKN2A
MDC1	XRCC1	FANCA	PMS1	ERCC4	POLD1		ERBB3		EP300	CYLD
RAD1		FANCB	PMS2	ERCC5 (alias xpg)	POLE	PI3KCA	ERBB4		B2M	CCND1
RAD17		FANCC	DNMT3A	ERCC6	POLE4	PIK3R1	EGFR		FOXL2	CCNE1
RAD9A		FANCD2		ERCC8	PRKDC	PPP2R1A	IGF1R			CDKN1A
RNF8		FANCE		RAD23A		PTEN	MET		WNT1	CDKN1B
		FANCF		XPA					CTNNB1	CDK16
		FANCG		XPC			THBS2		SMAD7	CDK17
		FANCI					PDGFRB		SMAD2	
		MUS81					FGFR2		Zeb1	
		NBP					FGFR3		APOBEC	
		PALB2					FGFR4		TOPO1	
		RAD50					FLT1		TOPO2B	
		RAD51					KDR		TOPO2A	
		RAD51C					FLT4		TOPBP1	
		RAD51D							ABCB1	
		RAD54L							TUBB3	
DNA Repair						Cell signaling			Miscellaneous	cell cycle
		XRCC2							BARD1	
		XRCC3							TAP1	
		SHFM1							TAP2	
									TAPBP	
									PALB2	
									CREBBP	
									RNF213	
									STK11	

**NGS SEQ GENE PANEL (159 genes)

Chemotherapy associated genes	Angiogenesis pathway	miRNA machinery	Immunogenetics and inflammation pathway	HLA gene family
APE1	VEGF-A	ADAR	CCL2	HLA-A
ERCC1	VEGF-B	Argonaute1 (EIF2C1)	CCL5	HLA-B
hMLH1	VEGF-C	Argonaute 2 (EIF2C2)	CCR2	HLA-C
hMSH2	VEGF-D	Argonaute4 (EIF2C4)	CCR7	HLA-E
hOGG 1	VEGF-E	CNOT1 (o NOT1)	CD274	HLA-F
RAD51	VEGF-F	CNOT2 (o NOT2)	CD276 (B7-H3)	HLA-G
XPD	IGF-1	CNOT3(o NOT3)	CTLA4	HLA-DP
XRCC1	IGF-2	CNOT4 (o NOT4)	CXCL12	HLA-DQ
XRCC3	IGFR-1	CNOT6 (o CCR4)	CXCL5	HLA-DR
CYP3A4	PIGF	DGCR8 (o Pasha)	CXCR3	
CYP3A5	VEGF-R1	Dicer	CXCR4	
CYP1B1	VEGF-R2	EDC4 (oHEDLS/Ge-1)	CXCR7	
CYP2C8	VEGF-R3	FMRP	FAS	
GST M1	PGF	GEMIN3 (o DDX20)	FOXO3	
GST T1	PDGFa	GEMIN4	FOXP3	
GSTM3	PDGFb	GEMIN5	IFNG	
GSTP1	PDGFc	HIWI	IFNGR1	
ABCG2	PDGFRa	hnRNP-A1	IFNGR2	
ABCC2	PDGFRb	LSM4	IL10RA	
ABCB1	NRP1	MOV10	IL10RB	
	HLFB	p68 (DDX5 DEAD)	IL12Rb1	
	HIF1α	POLR2A	IL13RA	
		Ran	IL15RA	
		RNASEN (Drosha)	IL17A	
		SMAD1	IL17F	
		SMAD2	IL17RA	
		SMAD3	IL18	
		SMAD5	IL1R1	
		SND1 (o Tudor-SN)	IL1R2	
		TNRC6A (o GW182)	IL1RN	
		TNRC6B	IL23R	
		TRBP	IL2RA	
		TUT4	IL2RB	
		XPO5 (exportin-5)	IL2RG	
			IL4R	
			IL6R	
			IL6ST	
			IL7R	
			IL8	
			IL8RA	
			ILR8B	
			LTA	
			MIF	
			MMp1	
			MMP13	
			MMP2	
			MMP3	
			MMp9	
			NFKb1	

			PDCPD1	

Chemotherapy associated genes	Angiogenesis pathway	miRNA machinery	Immunogenetics and inflammation pathway	HLA gene family
			PRDM1	
			PTGS2	
			SMAD2	
			SMAD3	
			SMAD4	
			SMAD7	
			STAT1	
			STAT3	
			STAT5A	
			STAT5B	
			STAT6	
			TGFBR1	
			TGFBR2	
			TIMP1	
			TIRAP	
			TLR10	
			TLR2	
			TLR3	
			TLR4	
			TLR6	
			TLR9	
			TNFRSF1A	
			TNFRSF1B	
			WNT5A	

Performing Labs:

G. CHIAPPETTA, S. SCALA, D. CALIFANO: Functional Genomic Unit, Istituto Nazionale per lo Studio e la Cura dei Tumori "Fondazione G. Pascale", IRCCS, Naples, Italy,
S. LOSITO, G. SCOGNAMIGLIO: Surgical Pathology Unit, Istituto Nazionale per lo Studio e la Cura dei Tumori "Fondazione G. Pascale", IRCCS, Naples, Italy
G. BALDASSARRE: Division of Experimental Oncology 2, Centro di Riferimento Oncologico, Istituto Ricovero e Cura Carattere Scientifico (CRO-IRCCS), Aviano, Italy
G. TOFFOLI, E. CECCHIN: Experimental and Clinical Pharmacology Unit, Centro di Riferimento Oncologico, Istituto Ricovero e Cura Carattere Scientifico (CRO-IRCCS), Aviano, Italy
G. SCAMBIA: Department of Obstetrics and Gynecology, Gynecologic Oncology Unit, Catholic University of the Sacred Heart, Rome, Italy
G. FERRANDINA: Division of Gynecologic Oncology, Catholic University of the Sacred Heart, Rome, Italy.
E. CAPOLUONGO: Laboratory of Clinical Molecµlar and Personalized Diagnostics, Department of Laboratory Medicine, "A Gemelli" Hospital, Catholic University, Rome, Italy.
GF. ZANNONI: Department of Human Pathology, Division of Gynecologic Pathology, Catholic University of the Sacred Heart, Rome, Italy
D. MEZZANZANICA: Molecµlar Therapies Unit, Dept of Experimental Oncology and Molecµlar Medicine, Fondazione IRCCS Istituto Nazionale dei Tumori Milan, Italy
S. INDRACCOLO: Immunology and Molecµlar Oncology Unit Istituto Oncologico Veneto - IRCCS Padova, Italy
S.FERRINI, M.FABBI: Department of Integrated Oncological Therapies, IRCCS AOU San Martino-IST Istituto Nazionale per la Ricerca sµl Cancro, Genoa, Italy
M. D'INCALCI, S.MARCHINI, R. GIAVAZZI: Department of Oncology IRCCS - Istituto di Ricerche Farmacologiche Mario Negri Milan, Italy
G. TOGNON, E. BIGNOTTI: Unit of Obstetrics and Gynecology-SPEDALI RIUNITI BRESCIA, Brescia, Italy
M. CAROSI: Division of Pathology Regina Elena Cancer Institute, Rome, Italy
MP. COSTI: Department of Life Sciences, University of Modena and Reggio Emilia, Modena, Italy.
F. BERTOLINI: Laboratory of Hematology-Oncology, European Institute of Oncology, Milan, Italy.
F. ESPOSITO: Department of Molecµlar Medicine and Medical Biotechnology, University of Naples Federico II, Naples, Italy.
C.Croce: Department of Molecular Virology, Immunology and Medical Genetics, The Ohio State University, Columbus, OH 43210
